# The Scattering of Phonons by Infinitely Long Quantum Dislocations Segments and the Generation of Thermal Transport Anisotropy in a Solid Threaded by Many Parallel Dislocations

**DOI:** 10.3390/nano10091711

**Published:** 2020-08-29

**Authors:** Fernando Lund, Bruno Scheihing-Hitschfeld

**Affiliations:** 1 Departamento de Física, Facultad de Ciencias Físicas y Matemáticas, Universidad de Chile, Santiago 8370449, Chile; bscheihi@mit.edu; 2 CIMAT, Facultad de Ciencias Físicas y Matemáticas, Universidad de Chile, Santiago 8370449, Chile; 3 Center for Theoretical Physics, Massachusetts Institute of Technology, Cambridge, MA 02139, USA

**Keywords:** thermal transport, dislocations, quantum field theory

## Abstract

A canonical quantization procedure is applied to the interaction of elastic waves—phonons—with infinitely long dislocations that can oscillate about an equilibrium, straight line, configuration. The interaction is implemented through the well-known Peach–Koehler force. For small dislocation excursions away from the equilibrium position, the quantum theory can be solved to all orders in the coupling constant. We study in detail the quantum excitations of the dislocation line and its interactions with phonons. The consequences for the drag on a dislocation caused by the phonon wind are pointed out. We compute the cross-section for phonons incident on the dislocation lines for an arbitrary angle of incidence. The consequences for thermal transport are explored, and we compare our results, involving a dynamic dislocation, with those of Klemens and Carruthers, involving a static dislocation. In our case, the relaxation time is inversely proportional to frequency, rather than directly proportional to frequency. As a consequence, the thermal transport anisotropy generated on a material by the presence of a highly-oriented array of dislocations is considerably more sensitive to the frequency of each propagating mode, and, therefore, to the temperature of the material.

## 1. Introduction

The search for efficient ways to transform waste heat into usable power has spurred research, both basic and applied, into thermoelectric materials wherein, for example, a temperature gradient generates an electric current. These phenomena involve the transport of energy and of electric charge, and are dominated by electrons and lattice vibrations: electrons carry both electric charge and energy, while phonons carry energy. The desire is then to optimize electron mobility (to obtain an “electron crystal”) while hampering as much as possible the motion of phonons (a “phonon glass”) [1]. In other words, obstacles should be put in the way of phonons that impede as little as possible the passage of electrons.

Within a polycrystal, obstacles to phonon motion include point defects—vacancies, interstitials, impurities—line defects such as dislocations, surface defects such as grain boundaries, interfaces, and free surfaces, and what could be termed three-dimensional defects such as precipitates. Recently, the role of dislocations has become the focus of much attention. For example, Shuai et al. [2] have reported high thermoelectric performance of Bi-based Zintl phases (Eu${}_{0.5}$Yb${}_{0.5}$)${}_{1-x}$Ca${}_{x}$Mg${}_{2}$Bi${}_{2}$, and have correlated this performance with an increase in the dislocation density of the material. In a similar vein, Wu et al. [3] have added small amounts of Na, Eu, and Sn to PbTe to obtain Na${}_{y}$Eu${}_{0.03}$Sn${}_{0.02}$Pb${}_{0.95-y}$ Te. They have shown that, when y=0.03, there is a significant increase of dislocation density, together with a significant decrease in thermal conductivity. You et al. [4] have studied the behavior of a PbSe-Cu system in which the presence of dislocations leads to a significantly decreased lattice thermal conductivity. Xin et al. [5] have studied the thermal behavior of Mg${}_{2}$Si${}_{1-x}$Sb${}_{x}$ and have concluded that an increase in dislocation density leads to a decrease in thermal conductivity. This strategy of introducing an extra alloying element into a given thermoelectric material has been followed by a number of researchers: Zhou et al. [6] have introduced Sb and Te into PbSe, while Yu et al. [7] introduced Ag into PbTe. In both cases, there was a decrease in the thermal conductivity that could be related to the presence of dislocations. A numerical experiment, using molecular dynamics, has been carried out by Giaremis et al. [8] in order to investigate the effect of decorated dislocations on the thermal conductivity of GaN. They have concluded that decorated dislocation engineering can lead to interesting fabrication strategies for themoelectric devices.

The theory tool used by the research mentioned in the previous paragraph is the classical analysis of Klemens [9]. In this work, dislocations are considered as static, straight line, defects of infinite length. That is, they are point defects in a two-dimensional lattice that extend themselves into the third dimension to plus and minus infinity by a homogeneous translation. Now, dislocations in any material have a finite length, a typical magnitude (except in especially designed materials, see below) being ∼100 nm or less. In addition, dislocations are by no means static. They respond to an incoming elastic wave, i.e., to a phonon, by bowing out, and this response has been well-known and widely documented over decades [10,11,12]. Admittedly, there is no denying that the data mentioned in the previous paragraph can be fit with a contribution to the phonon inverse relaxation time that is a linear combination of terms linear in frequency and cubic in frequency, as explained by Klemens. However, there does not appear to be a clear relation between the parameters needed to obtain a fit to the data and the parameters characterizing each specific material. From a different perpective, Wang et al. have performed ab initio numerical calculations of the scattering of phonons by dipole dislocations in GaN [13] and in Si [14] concluding that, while said scattering is significant, it is not quantitatively accounted for by the model of Klemens [9]. Clearly, a better modeling of the phonon-dislocation interaction is needed. In this paper, we address this concern.

A rather significant development has been reported by Sun et al. [15], who have fabricated a micron thick InN single crystal with a highly oriented dislocation array that pierces through the film across, and have measured the thermal conductivity both parallel and perpendicular to the film thickness. There is a factor as high as ten between the two, which is far larger than what would be expected from the single crystal anisotropy. Of course, free boundaries and point defects are the same for both directions. This anisotropy has been measured as a function of temperature and of dislocation density. The model of Klemens [9] can reproduce, roughly, the temperature dependence of the cross-plane thermal conductivity, but cannot reproduce its dependence on dislocation density; its prediction is also quite far away from the observed values for the in-plane thermal conductivity.

A modification of the model of Klemens [9] was worked out by Carruthers [16] in order to provide a theoretical basis for a larger phonon-dislocation scattering cross section. While the theory of Carruthers [16] provides a crude estimate of the results of Sun et al. [15], it does not appear to accurately capture the temperature dependence, or the dislocation dependence, of the complete thermal conductivity tensor (involving both in- and cross-plane conductivities). In any case, the calculation of Carruthers [16] is based on an anharmonic interaction between atoms that does not appear to have received independent validation.

An additional feature of the experiment of Sun et al. [15] is that most heat-carrying phonons appear to have wavelengths shorter than 5 nm, which is much smaller than the apparent typical length of a dislocation in their setup, L∼1μm. Therefore, understanding the interaction between phonons and dislocations where the wavelength of the former is much smaller than the spatial extent of the latter should prove to be essential in describing and explaining thermal transport anisotropies mediated by dislocations. In a previous publication [17], we have developed a quantum theory of phonons in interaction with dislocation segments of finite length. Here, we extend that formalism to infinitely long dislocations, and explore the consequences for the thermal transport properties of a material threaded with such many parallel dislocations.

The dynamic response of an infinitely long dislocation to an incoming phonon was considered by Ninomiya [18,19], who introduced a phonon–dislocation coupling through the kinetic energy terms in the system Hamiltonian, while not retaining a potential energy coupling. This interaction has been recently quantized by Li et al. [20,21,22]. In this paper, we shall quantize the dislocation–phonon interaction (DPI) when the dislocation is an infinitely long elastic string, but the coupling, as explained in more detail in the next section, is through the potential energy term and is chosen to reproduce well-established classical results, as encapsulated by the Peach–Koehler force [12,23].

The possibility that oscillating—as opposed to static—dislocations should contribute to thermal transport was considered by Granato [24] soon after the work of Klemens [9]. However, no satisfactory agreement with experimental measurements could be found. In retrospect, it would appear that one difficulty with the theory, as gleaned from the 1982 review by Kneezel and Granato [25], was that the phonon scattering rate induced by the moving dislocations was calculated to be proportional to a damping term in the dislocation dynamics, itself proportional to dislocation velocity, with an uncontrolled proportionality constant. In the present paper, we introduce no such phenomenological parameters. On the contrary, our theoretical framework sets the stage for the calculation of velocity-dependent damping of long wavelength phonons by their interaction with short wavelength dislocation oscillations. We shall briefly touch on this issue below.

This paper is organized as follows: Section 2 recalls the classical theory that will be quantized. Section 3 introduces the canonical quantization of the free phonons and free dislocation modes which, following Li et al. [20], we shall call “dislons”. Section 4 introduces the interaction between phonons and dislons and presents a number of consequences of this interaction, calculated to lowest order (classically, this means for small strains). The basic tools are described, and the T matrix for the scattering of a phonon by a dislocation is calculated in Section 4.1, while Section 4.2 discusses how the particle-like properties of dislons are modified by their interaction with phonons. An interesting result is the computation of the phonon contribution to the phenomenological damping (the so-called “*B*” term) that is introduced in classical descriptions of dislocation dynamics as elastic strings [10,26]. A number of computations are presented in two Appendices. The consequences of the aforementioned interaction for thermal transport are worked out in Section 5, with special attention to the case of a solid threaded by a large number of parallel dislocations. Phonon scattering cross sections and lifetimes are discussed in Section 5.1 and Section 5.2, respectively. Section 5.3 compares the consequences with the classical results of Klemens and Carruthers. One striking difference is that, while in the Klemens/Carruthers approach, the phonon lifetime is proportional to frequency and, if the contribution of the dislocation core is considered, to frequency cubed, our approach leads to a phonon lifetime that is *inversely proportional* to frequency, as discussed in Section 5.3. A quantitative comparison of the resulting anisotropy in thermal transport is given in Section 5.4. Finally, Section 6 has a discussion, outlook, and concluding remarks.

## 2. Classical Action

In this work, we work out the quantum theory of oscillating dislocation segments, of infinite length, in interaction with elastic waves in three dimensions. The quantum theory to be constructed is based on a well-established classical theory, whose principal aspects we recap here.

We consider a homogeneous, solid, elastic continuum of density ρ, possibly anisotropic along one axis ${\hat{e}}_{3}$, and elastic constants $c_{pqmr}\;\left(p,q=1,2,3\right)$. Within the solid, there is a string-like dislocation line. The variables describing the solid are the displacements u(x,t), at time *t*, of a point whose equilibrium position is x. The string is described by a vector X(s,t), where −∞<s<∞ is the coordinate along the string equilibrium axis, whose endpoints are fixed at spatial infinity. The motion of this string is one of small amplitude away from an equilibrium position that is a static straight line parametrized by $X_{0}\left(s\right)=s\;{\hat{e}}_{3}$. The fact that the string is a dislocation is implemented by the displacements u(x,t) being multivalued functions: they have a discontinuity equal to the Burgers vector b when crossing a surface whose boundary is the string. In addition to this geometrical fact, the coupling between elastic displacements and elastic string is given by standard conservation of energy and momentum arguments [27]. When dislocation velocities are small compared to the speed of sound, an assumption we shall make throughout this work, this leads to the well-known Peach–Koehler force [23]. In the time-dependent case, and for string velocities small compared to the speed of sound, the dynamics are described by the following classical action:

(1) $$S=S_{\mathrm{ph}}+S_{\mathrm{string}}+S_{\mathrm{int}}+S_{0}$$

where

(2) $$\begin{array}{ccc} S_{\mathrm{ph}} & = & \frac{1}{2}\int \;dt\;\int \;d^{3}x\;\left(\rho {\dot{u}}^{2}-c_{pqmr}\frac{\partial u_{m}}{\partial x_{q}}\frac{\partial u_{p}}{\partial x_{r}}\right)+\cdots \end{array}$$

(3) $$\begin{array}{ccc} S_{\mathrm{string}} & = & \frac{1}{2}\int \;dt\;\int_{-\infty }^{\infty }\;\;\;ds\;\left(m{\dot{X}}^{2}-\Gamma X^{\prime 2}\right)+\cdots \end{array}$$

(4) $$\begin{array}{ccc} S_{\mathrm{int}} & = & -b_{i}\int \;dt\;\int_{\delta S}dS^{j}\sigma_{ij}. \end{array}$$

where the ellipses “⋯” refer to higher order terms in the phonon or string actions in the sense that they involve higher powers of the dynamic fields u and X. In addition, $b_{i}$ is the *i*-th component of the Burgers vector, $\sigma_{ij}$ is the elastic stress tensor, evaluated at the current position of the dislocation line, and the surface δS describes the region bounded by the string and its equilibrium position. Finally, $S_{0}$ involves the interaction of the elastic displacements with a static dislocation, whose boundary is the straight line $X_{0}$. It does not contribute to the dynamics and shall be ignored in the sequel.

Let us start by describing the phonon action $S_{\mathrm{ph}}$. Clearly, $S_{\mathrm{ph}}$ describes elastic waves in an elastic continuum, wherein the quadratic terms will lead to free phonons and the higher order terms will lead to phonon–phonon interactions. In the isotropic case, $c_{pqmr}=\lambda \delta_{pq}\delta_{mr}+\mu \left(\delta_{pm}\delta_{qr}+\delta_{pr}\delta_{mq}\right)$ where λ and μ are the Lamé constants. However, in the axially anisotropic case, for a medium with hexagonal or transverse isotropic symmetry, there are five independent elastic constants.

In the absence of the interaction term, the free phonon theory has simple solutions in terms of plane waves for the elastic displacement (phonons) and normal modes for the elastic string-like dislocation. In the case of phonons, they are in general characterized by three different modes of propagation, determined by the wave equation

(5) $$\rho {\ddot{u}}_{p}=c_{pqmr}\frac{\partial^{2}u_{m}}{\partial x_{r}\partial x_{q}}.$$

In the completely isotropic case, we have only two distinct modes: transversal waves of speed $c_{T}=\sqrt{\mu /\rho }$ with two allowed polarizations, and longitudinal waves of speed $c_{L}=\sqrt{(\lambda +2\mu )/\rho }$ with one polarization.

On the other hand, we have a string described by a vector field X(s,t), where −∞<s<∞ is a position parameter along said string. We consider small deviations from a straight equilibrium position $X_{0}$, the ends of which are pinned to their positions at infinity. $S_{\mathrm{string}}$ describes oscillations (normal modes) of an elastic string of infinite length with fixed ends; higher order terms describe anharmonic effects on these oscillations, which we will not address in this work. The parameters *m* (mass per unit length) and Γ (line tension) characterize the dislocation segment.

In previous work [17], considering only the completely isotropic case, we have assumed segments of edge dislocations only, in which case they may be written in terms of the Burgers vector b and $\gamma \equiv c_{L}/c_{T}$ as

(6) $$m_{\mathrm{isotropic}}=\frac{\rho b^{2}}{4\pi }\left(1+\gamma^{-4}\right)\ln\left(\delta /\delta_{0}\right)$$

where δ, $\delta_{0}$ are long- and short-distance cutoff lengths, and

(7) $$\Gamma_{\mathrm{isotropic}}=\frac{\mu b^{2}}{2\pi }\left(1-\gamma^{-2}\right)\ln\left(\delta /\delta_{0}\right).$$

For an anisotropic solid, one can expect that the factors $\left(1+\gamma^{-4}\right)$ and $\left(1-\gamma^{-2}\right)$ in *m* and Γ will be modified to reflect the specific geometry of the solid, but should otherwise remain unchanged.

Classically, phonons have plane wave solutions. Similarly, the string term $S_{\mathrm{string}}$ of the action leads to oscillatory solutions that may be expanded in Fourier series Re∫dκ2πa(κ)e−iωκte−iκs, where $\omega_{\kappa }=\kappa \sqrt{\frac{\Gamma }{m}}$ is the frequency of each normal mode. We take the string to have one degree of freedom (i.e., one direction orthogonal to its equilibrium position over which to oscillate) defined by the direction of the Burgers vector b, thus defining the glide plane. For most of our results, the generalization to more directions of oscillation is straightforward.

Finally, $S_{\mathrm{int}}$ describes the interaction between these two sectors. It is straightforward to check that

(8) $$\frac{\delta S_{\mathrm{int}}}{\delta X_{k}}=-b_{i}\varepsilon_{jkm}X_{m}^{\prime }\sigma_{ij}$$

reproduces the well known Peach–Koehler force [12,23]. This DPI coupling has been successfully used for decades [10,11]. In recent years, it has been used to compute the scattering cross section of elastic waves by dislocation segments in a first Born approximation, a result that has been further employed to compute the change in propagation velocity and attenuation for said waves by many such dislocation segments [28,29,30]. These results, in turn, have led to novel ways of acoustically characterizing the plasticity of metals and alloys [31,32,33,34].

Having reviewed the classical theory, we now turn to its quantization by introducing canonical commutation relations. We note that the theoretical setup just described includes anisotropic media. Even though we shall work out specific quantitative consequences only in the isotropic case, we shall keep the notation, as far as possible, compatible with anisotropy.

## 3. Canonical Quantization of the Free Fields

We commence this section by implementing the correspondence of Poisson brackets to commutators $\left\{\cdot ,\cdot \right\}\to -\frac{i}{\hbar }\left[\cdot ,\cdot \right]$. According to standard practice [35,36], the mode coefficients of the classical solutions are promoted to creation and annihilation operators, in terms of which we may write the displacement field as

(9) $$u\left(x,t\right)=\sqrt{\frac{\hbar }{\rho }}\;\int \;\frac{d^{3}k}{{\left(2\pi \right)}^{3}}\;\sum_{\iota \in \{\mathrm{pol}.\}}\left[\frac{\varepsilon_{\iota }^{*}\left(k\right)a_{\iota }\left(k\right)e^{ik\cdot x-i\omega_{\iota }\;\left(k\right)t}}{\sqrt{2\omega_{\iota }\;\left(k\right)}}+\frac{\varepsilon_{\iota }\left(k\right)a_{\iota }^{†}\left(k\right)e^{-ik\cdot x+i\omega_{\iota }\;\left(k\right)t}}{\sqrt{2\omega_{\iota }\;\left(k\right)}}\right],$$

where the sum over ι represents the sum over phonon polarizations. On the other hand, we may write the string displacement as

(10) $$X\left(s,t\right)=\sqrt{\frac{\hbar }{m}}\int \frac{d\kappa }{2\pi }\left(\frac{\alpha \left(\kappa \right)e^{-i\omega_{\kappa }t}e^{-i\kappa s}}{\sqrt{2\omega_{\kappa }}}+\frac{\alpha^{†}\left(\kappa \right)e^{i\omega_{\kappa }t}e^{i\kappa s}}{\sqrt{2\omega_{\kappa }}}\right).$$

Now, we proceed to impose canonical commutation relations:

(11) $$\begin{array}{ccc} \left[u_{i}\left(x,t\right),\rho {\dot{u}}_{j}\left(y,t\right)\right] & = & i\hbar \delta_{ij}\delta^{\left(3\right)}\left(x-y\right), \end{array}$$

(12) $$\begin{array}{ccc} \left[X\left(s,t\right),m\dot{X}\left(s^{\prime },t\right)\right] & = & i\hbar \delta (s-s^{\prime }), \end{array}$$

(13) $$\begin{array}{ccc} {}[u_{i}\left(x,t\right),u_{j}\left(y,t\right)]=0, & & [X\left(s,t\right),X\left(s^{\prime },t\right)]=0, \end{array}$$

which fully define the quantum theory in the non-interacting case. These relations in turn require

(14) $$\begin{array}{cc} \left[a_{\iota }\left(k\right),a_{\iota^{\prime }}\left(k^{\prime }\right)\right] & =[a_{\iota }^{†}\left(k\right),a_{\iota^{\prime }}^{†}\left(k^{\prime }\right)]=0, \end{array}$$

(15) $$\begin{array}{cc} {}[a_{\iota }\left(k\right),a_{\iota^{\prime }}^{†}\left(k\right)] & ={\left(2\pi \right)}^{3}\delta^{\left(3\right)}\left(k-k^{\prime }\right)\delta_{\iota \iota^{\prime }}, \end{array}$$

(16) $$\begin{array}{cc} {}[\alpha \left(\kappa \right),\alpha \left(\kappa^{\prime }\right)] & =[\alpha^{†}\left(\kappa \right),\alpha^{†}\left(\kappa^{\prime }\right)]=0, \end{array}$$

(17) $$\begin{array}{cc} {}[\alpha \left(\kappa \right),\alpha^{†}\left(\kappa^{\prime }\right)] & =\left(2\pi \right)\delta^{\left(1\right)}\left(\kappa -\kappa^{\prime }\right). \end{array}$$

In the preceding expressions, ι is an index that runs over the possible polarizations for the phonons, which in the isotropic case goes over two transverse polarizations that we will denote by $\iota =T_{1},T_{2}$, and one longitudinal polarization that we will denote by ι=L. In the anisotropic case, there would be three inequivalent polarizations: transverse polarization with displacements within the ${\hat{e}}_{1}-{\hat{e}}_{2}$ plane, transverse polarization within the ${\hat{e}}_{3}-\hat{k}$ plane, and longitudinal polarization inside the ${\hat{e}}_{3}-\hat{k}$ plane. $\varepsilon_{\iota }\left(k\right)$ represents the polarization vector associated with each mode of propagation. The corresponding eigenfrequencies satisfy $\omega_{\iota }\left(k\right)=c_{\iota }\left(\hat{k}\cdot {\hat{e}}_{3}\right)k$, with two phase velocities $c_{T},c_{L}$ in the isotropic case, and three phase velocities that depend on the angle between the wave-vector and the anisotropy axis in the anisotropic case. Finally, $\omega_{\kappa }=\kappa \sqrt{\Gamma /m}$ is the frequency for the mode of the string with wavenumber κ.

To complete the description of the theory, we need to specify its dynamics, which are generated by the time-evolution implied by a Hamiltonian operator. In the case of the “free” theory, where no interactions between phonons and “dislons” (the excitations on the string) take place, the Hamiltonian, obtained from the action (1) by the usual canonical transformation, is

(18) $$H=H_{\mathrm{ph}}+H_{\mathrm{string}}$$

with phonon and string terms given, respectively, by

(19) $$\begin{array}{ccc} H_{\mathrm{ph}} & = & \int \;\frac{d^{3}k}{{\left(2\pi \right)}^{3}}\;\sum_{\iota \in \{\mathrm{pol}.\}}\hbar \omega_{\iota }\left(k\right)a_{\iota }^{†}\left(k\right)a_{\iota }\left(k\right), \end{array}$$

(20) $$\begin{array}{ccc} H_{\mathrm{string}} & = & \int \frac{d\kappa }{2\pi }\hbar \omega_{\kappa }\alpha^{†}\left(\kappa \right)\alpha \left(\kappa \right). \end{array}$$

In characterizing the free theory, a fundamental object is the two-point function, more commonly known as the *propagator*. Let T be the time-ordering symbol, instructing operators evaluated at a later time to be placed at the left, and let |0〉 be the vacuum state of the quantum mechanical system, with no excitations of the elastic displacements nor the string. For the dislocation, it reads

(21) $$\begin{array}{cc} \Delta (s-s^{\prime },t-t^{\prime })\equiv & \langle 0|TX\left(s,t\right)X\left(s^{\prime },t^{\prime }\right)|0\rangle \\ = & \frac{\hbar }{m}\int \frac{d\kappa }{2\pi }\frac{e^{-i\omega_{\kappa }\left|t-t^{\prime }\right|}}{2\omega_{\kappa }}e^{i\kappa (s-s^{\prime })}. \end{array}$$

Even though we can do the same for the elastic displacement field, it turns out to be more useful for subsequent computations to write down the propagator for its spatial derivative:

(22) $$\begin{array}{cc} \Delta_{iji^{\prime }j^{\prime }}\left(x-x^{\prime },t-t^{\prime }\right)\equiv & \;\langle 0|T\frac{\partial u_{i}}{\partial x_{j}}\left(x,t\right)\frac{\partial u_{i^{\prime }}}{\partial x_{j^{\prime }}}\left(x^{\prime },t^{\prime }\right)|0\rangle \\ = & \frac{\hbar }{\rho }\int \frac{d^{3}k}{{\left(2\pi \right)}^{3}}\sum_{\iota \in \{\mathrm{pol}.\}}k_{j}k_{j^{\prime }}\varepsilon_{\iota }{\left(\hat{k}\right)}_{i}\varepsilon_{\iota }^{*}{\left(\hat{k}\right)}_{i^{\prime }}\frac{e^{-i\omega_{\iota }\left(k\right)\left|t-t^{\prime }\right|}}{2\omega_{\iota }\left(k\right)}e^{ik\cdot (x-x^{\prime })}\\ = & \frac{\hbar }{\rho }\int \frac{d^{3}k}{{\left(2\pi \right)}^{3}}\int \frac{d\omega }{2\pi i}\sum_{\iota \in \{\mathrm{pol}.\}}\frac{k_{j}k_{j^{\prime }}\varepsilon_{\iota }{\left(\hat{k}\right)}_{i}\varepsilon_{\iota }^{*}{\left(\hat{k}\right)}_{i^{\prime }}}{-\omega^{2}+\omega_{\iota }{\left(k\right)}^{2}-i\epsilon }e^{ik\cdot (x-x^{\prime })}e^{-i\omega (t-t^{\prime })}, \end{array}$$

where ϵ is a positive infinitesimal.

The reason behind writing down time-ordered quantities is that, when we compute scattering amplitudes in the interacting theory, we will be interested in the *S*-matrix, given by [35,36]

(23) $$\langle \Psi_{\mathrm{out}}\left|T\exp\left[-\frac{i}{\hbar }\int_{-\infty }^{\infty }H_{I}\left(t\right)dt\right]\;\right|\Psi_{\mathrm{in}}\rangle$$

where $H_{I}$ is the quantum mechanical interaction picture Hamiltonian operator. Therefore, if we expand the exponential in (23) in a power series of $H_{I}$, all terms in the series will be time-ordered, thus giving the computation of time-ordered quantities a central role.

In what follows, we will denote by $u^{I}$ and $X^{I}$ the operator fields associated with lattice displacements and to the oscillations of the string-like dislocation, respectively, in the interaction picture of quantum mechanics, which evolve as free fields. As a reminder to the reader, the passage between the interaction picture and the Heisenberg picture is implemented through

(24) $$u\left(x,t\right)=U^{†}\left(t,t_{0}\right)u_{I}\left(x,t\right)U\left(t,t_{0}\right)$$

with

(25) $$U\left(t,t_{0}\right)=T\exp\left[-i\int_{t_{0}}^{t}H_{I}\left(t^{\prime }\right)dt^{\prime }\right]$$

where $t_{0}$ is the time at which both operators coincide (typically in a scattering context, it is taken to be −∞).

Having set up the formalism, we can now dive into the interaction Hamiltonian $H_{I}$ of interest and explore the dynamics it generates for the constituents of our theory: phonons and dislons.

## 4. The Quadratic Interactions with a Single String

The interaction term $S_{\mathrm{int}}$ (4) is an infinite series in increasing order of gradients of the particle displacement. The lowest order interaction, which classically means to consider small strains and small string excursions away from the equilibrium position, is given by

(26) $$S_{\mathrm{int}}^{\left(2\right)}=-Nb\int dt\int_{-\infty }^{\infty }\;\;\;\;ds\;M_{kl}\frac{\partial u_{k}}{\partial x_{l}}\left(x_{0}+\left(0,0,s\right),t\right)X\left(s,t\right)$$

which is quadratic in the fluctuations. In this expression, we have defined $N\equiv c_{1212}$. We will take the string to have its equilibrium position along the ${\hat{e}}_{3}$ axis, and the Burgers vector to be written as $b=b{\hat{e}}_{1}$. With this choice of coordinates, $M_{kl}={\left({\hat{e}}_{1}\right)}_{k}{\left({\hat{e}}_{2}\right)}_{l}+{\left({\hat{e}}_{2}\right)}_{k}{\left({\hat{e}}_{1}\right)}_{l}$. This interaction will give rise to the scattering of phonons by the string, which is described by

(27) $$\langle f|i\rangle =\langle 0|a_{\iota^{\prime }}\left(k^{\prime }\right)T\exp\left[-\frac{i}{\hbar }\int_{-\infty }^{\infty }H_{I}\left(t\right)dt\right]a_{\iota }^{†}\left(k\right)|0\rangle ,$$

where

(28) $$H_{I}\left(t\right)=Nb\int_{-\infty }^{\infty }\;\;\;\;ds\;M_{kl}\frac{\partial u_{k}^{I}}{\partial x_{l}}\left(x_{0}+\left(0,0,s\right),t\right)X^{I}\left(s,t\right),$$

and $a_{\iota }^{†}\left(k\right)$, $a_{\iota^{\prime }}\left(k^{\prime }\right)$ are creation and annihilation operators that define the initial (one phonon with wavenumber k and polarization ι) and final (one phonon with wavenumber $k^{\prime }$ and polarization $\iota^{\prime }$) states.

For completeness, we also write down the Hamiltonian in the Heisenberg picture (where it is naturally constant) in terms of creation and annihilation operators:

(29) $$H_{\mathrm{int}}=\hbar \int \frac{d^{3}k}{{\left(2\pi \right)}^{3}}\sum_{\iota \in \{\mathrm{pol}.\}}\left(\frac{iE\left(k;\iota \right)a_{\iota }\left(k\right)}{\sqrt{2\omega_{k_{3}}}}\left(\alpha \left(-k_{3}\right)+\alpha^{†}\left(k_{3}\right)\right)+h.c.\right)$$

where h.c. stands for Hermitian conjugate. Note that this is a quadratic interaction. Furthermore, following previous work [17], we have defined

(30) $$E\left(k;\iota \right)\equiv \frac{Nb}{\sqrt{2\rho m\omega_{\iota }\left(k\right)}}k_{l}M_{kl}\varepsilon_{\iota }^{*}{\left(k\right)}_{k}e^{ix_{0}\cdot k},$$

and we will denote its complex conjugate by $E^{*}$. Here, we have denoted $k_{l}=\left(k\cdot {\hat{e}}_{l}\right)$ and $\varepsilon_{\iota }{\left(k\right)}_{k}=\left(\varepsilon_{\iota }\left(k\right)\cdot {\hat{e}}_{k}\right)$.

We now turn to the main question of interest in this article: how does a (comparatively) short wavelength phonon with wavenumber k and polarization ι propagating through the elastic continuum interact with a long (approximately infinite) dislocation segment with length *L*? To answer this question, we proceed as follows: In Section 4.1, we organize and solve the theory in terms of Feynman diagrams, to then study the properties of dislons as particles and scatterers in Section 4.2. Finally, in Section 4.3, we explore the full phonon propagator in the presence of a single dislocation line and comment on its relevant features, before moving on to the implications of this interaction on thermal transport in the subsequent section. For completeness, we briefly discuss how the DPI makes both excitations reach thermal equilibrium in Appendix B.

### 4.1. Phonon by Dislocation Scattering: Amplitudes and Feynman Diagrams

In this section, we describe how to obtain the scattering amplitude of a phonon by a dislocation, to all orders in the interaction Hamiltonian (28). That is, we explicitly perform the computation of all terms in the power series development of the exponential in (27). Since we have a quantum field theory in our hands, it is natural to carry out the computation in terms of Feynman diagrams. This is a powerful method to organize the various terms that appear in scattering processes.

**Figure 1:**
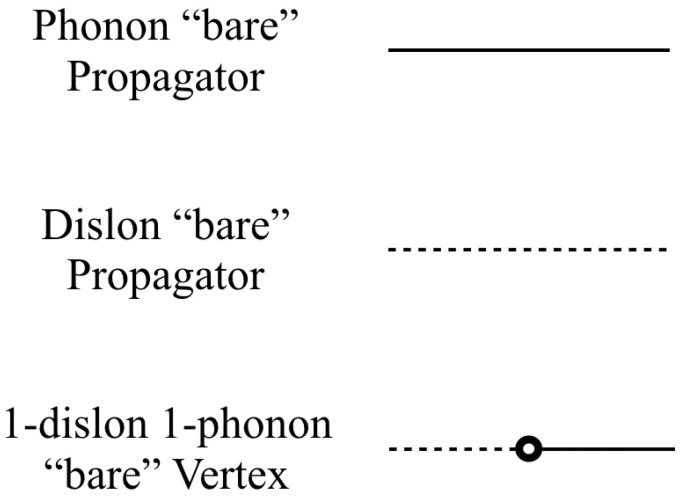
The basic diagrammatic expressions to be used in solving the quadratic theory. The upper two diagrams describe the basic propagator (Green’s function) for each type of excitation in the solid, with a continuous line for phonons and a dashed line for dislons. The third diagram represents the quadratic phonon–dislon interaction, allowing for quantum of excitations of one type to be converted into the other.

In the quadratic theory, the basic diagrammatic elements are those shown in Figure 1, each representing a specific contribution that gives form to the scattering processes. They are:

- Phonon “bare” propagator: the phonon Green’s function. In particular, we will be more interested in its derivatives, or the earlier defined $\Delta_{iji^{\prime }j^{\prime }}\left(x-x^{\prime },t-t^{\prime }\right)$ whose value in momentum–frequency space is given by
  (31) $$\Delta_{iji^{\prime }j^{\prime }}\left(k,\omega \right)=\sum_{\iota }\frac{-i\hbar k_{j}k_{j^{\prime }}\varepsilon_{\iota }{\left(\hat{k}\right)}_{i}\varepsilon_{\iota }^{*}{\left(\hat{k}\right)}_{i^{\prime }}}{-\rho \omega^{2}+\rho c_{\iota }^{2}\left(\hat{k}\right)k^{2}-i\epsilon },$$
  where we have omitted an overall Dirac delta imposing momentum–frequency conservation ${\left(2\pi \right)}^{4}\delta \left(\omega -\omega^{\prime }\right)\delta^{3}\left(k-k^{\prime }\right)$.
- Dislon “bare” propagator: the dislon Green’s function. It is given in momentum–frequency space by
  (32) $$\Delta \left(\kappa ,\omega \right)=\frac{-i\hbar }{-m\omega^{2}+\Gamma \kappa^{2}-i\epsilon },$$
  where we have also omitted an overall Dirac delta imposing momentum–frequency conservation ${\left(2\pi \right)}^{2}\delta \left(\omega -\omega^{\prime }\right)\delta \left(\kappa -\kappa^{\prime }\right)$.
- 1-dislon 1-phonon “bare” vertex: diagrammatical representation of the quadratic interaction between phonons and dislons. In position space, it instructs to operate over the coincident position coordinate of both adjacent propagators as
  (33) $$iV_{2}=\frac{-ibc_{1212}M_{i^{\prime }j^{\prime }}}{\hbar }\int_{-\infty }^{\infty }ds\Delta_{iji^{\prime }j^{\prime }}(x-\left(0,0,s\right),t-t^{\prime })\Delta (s-s\prime ,t^{\prime },-t^{\prime \prime }).$$
  This coupling will have important consequences when we examine the exact propagator for the dislocation excitations. Moreover, it enforces momentum (wavenumber) conservation along the direction of the dislocation line, through a factor $\left(2\pi \right)\delta \left(\kappa -k_{3}^{\prime }\right)$ that appears after integrating over the string coordinate *s*.

**Figure 2:**
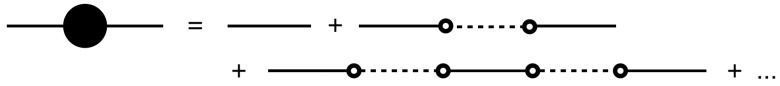
“Dressed” phonon propagator. This is the main composite object that appears in the quadratic theory calculations, and it does so as a consequence of incorporating the consequences of the interaction to all orders in perturbation theory.

With these tools, we want to evaluate (27), which corresponds to having one ingoing phonon and one outgoing phonon as external states, which in the diagrams that represent our scattering process are depicted by the “external” lines (i.e., those that have one of their ends not attached to another diagrammatic piece). In essence, we want to compute a “dressed” propagator for the elastic displacement field, which determines the probability of measuring a phonon with wave-vector $k^{\prime }$ as a result of having sent in a phonon with momentum k into the elastic medium. This is schematically represented in Figure 2.

The computation can now be organized by the number of vertices (i.e., insertions of the interaction-picture Hamiltonian as a result of expanding the time evolution operator in a power series) present in each diagrammatic piece of Figure 2. By the means of Wick’s theorem [35,36], one can then evaluate the expectation value corresponding to the transition amplitude (27), which essentially contracts all interaction-picture fields in pairs in all possible ways amongst themselves.

**Figure 3:**
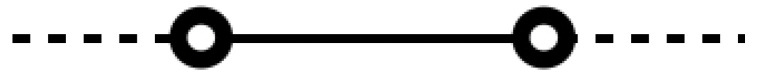
Diagrammatic representation of the dislon self-energy Π, where the dashed “external” lines carry ingoing and outgoing momenta κ and frequency ω.

This contraction in all possible ways effectively gives an n! factor that cancels the 1/n! in the Taylor expansion of the exponential because the time-ordering symbol makes all contractions equivalent at a fixed order in the perturbative series. Furthermore, phonon propagators conserve 3-momentum (the wavenumber k) and frequency/energy ω, whereas dislon propagators only conserve momentum along the dislocation axis $k_{3}\equiv k\cdot {\hat{e}}_{3}$ and energy ω. Then, since the integrals in the interaction-picture time evolution operator (one over time and one over the longitudinal extent of the dislocation *s*) propagate the conservation of $k_{3}$ and ω, all one needs to do is work out the pieces in the diagram in Figure 2 where modes with $k_{1}$ and $k_{2}$ components appear as “virtual” intermediate states. This essentially amounts to calculating the *self-energy*Π(κ,ω) of the dislon propagator, as is depicted in Figure 3. After evaluating this quantity, the remainder of the computation will be given by summing over the number of intermediate dislon propagators to construct the *exact* dislon propagator,

(34) $$\begin{array}{cc} S(\kappa ,\omega ) & \equiv \Delta \left(\kappa ,\omega \right)\sum_{n=1}^{\infty }{\left(\frac{i}{\hbar }\Pi \left(\kappa ,\omega \right)\Delta \left(\kappa ,\omega \right)\right)}^{n-1}\\ & =\frac{1}{\Delta^{-1}\left(\kappa ,\omega \right)-\frac{i}{\hbar }\Pi \left(\kappa ,\omega \right)}, \end{array}$$

which includes the effect of all the intermediate phonon states in a scattering process.

Using the diagrammatic elements we defined earlier, and letting (κ,ω), $\left(\kappa^{\prime },\omega^{\prime }\right)$ label the ingoing and outgoing states respectively, we obtain the following expression for the dislon self-energy

(35) $$\begin{array}{cc} & \left(2\pi \right)\delta \left(\omega -\omega^{\prime }\right)\left(2\pi \right)\delta \left(\kappa -\kappa^{\prime }\right)\frac{i}{\hbar }\Pi \left(\kappa ,\omega \right)\\ & =\frac{{\left(-i\right)}^{2}N^{2}b^{2}}{\hbar^{2}}M_{kl}M_{k^{\prime }l^{\prime }}\;\;\int_{-\infty }^{\infty }\;\;\;\;\;dt\int_{-\infty }^{\infty }\;\;\;\;\;dt^{\prime }\int_{-\infty }^{\infty }\;\;\;\;\;ds\int_{-\infty }^{\infty }\;\;\;\;\;ds^{\prime }\int \;\;\frac{d^{3}k}{{\left(2\pi \right)}^{3}}\;\;\int \frac{d\omega^{\prime \prime }}{2\pi i}\\ & \;\;\;\;\;\;\;\;\;\times \Delta_{klk^{\prime }l^{\prime }}\left(k,\omega \right)e^{i\left(s-s^{\prime }\right)k_{3}}e^{-i\omega^{\prime \prime }\left(t-t^{\prime }\right)}e^{i\omega t-i\omega^{\prime }t^{\prime }}e^{-i\kappa s+i\kappa^{\prime }s^{\prime }}. \end{array}$$

The integrals over time and $\omega^{\prime \prime }$ are straightforward and give energy conservation. Some algebra allows one for computing the integral over the azimuthal angle and obtain

(36) $$\begin{array}{cc} & \left(2\pi \right)\delta \left(\kappa -\kappa^{\prime }\right)\Pi \left(\kappa ,\omega \right)\\ & \;=\frac{N^{2}b^{2}}{16\pi^{2}\rho }\int_{-\infty }^{\infty }\;\;\;\;\;ds\int_{-\infty }^{\infty }\;\;\;\;\;ds^{\prime }\int_{-\infty }^{\infty }\;\;\;\;\;dk\int_{-1}^{1}\;\;\;due^{iku(s-s^{\prime })}e^{-i\kappa s+i\kappa^{\prime }s^{\prime }}k^{4}\left(1-u^{2}\right)\sum_{\iota }\frac{|\varepsilon_{\iota }^{xy}{\left(u\right)|}^{2}}{c_{\iota }^{2}\left(u\right)k^{2}-\omega^{2}-i\epsilon }, \end{array}$$

where u≡cosθ and we have extended the radial integral over *k* to −∞ as the integrand is symmetric under k→−k and u→−u. In this equation, we have introduced the quantity $|\varepsilon_{\iota }^{xy}\left(u\right)|$, which stands for the magnitude of the projection of the polarization vector $\varepsilon_{\iota }$ into the ${\hat{e}}_{1}-{\hat{e}}_{2}$ plane.

From here, one proceeds to evaluate the integral over *k* by contour integration, closing on the upper-half plane if $u(s-s^{\prime })>0$, and on the lower-half plane otherwise. After a straightforward, but tedious calculation, one arrives at

(37) $$\Pi \left(\kappa ,\omega \right)=m\omega^{2}F\left(\kappa /\omega \right),$$

where the function *F* is given by

(38) $$F\left(x\right)=\frac{N^{2}b^{2}}{8\pi \rho m}\sum_{\iota \in \{\mathrm{pol}.\}}\int_{0}^{1}du\frac{|\varepsilon_{\iota }^{xy}{\left(u\right)|}^{2}}{c_{\iota }^{4}\left(u\right)}\frac{2u(1-u^{2})}{x^{2}c_{\iota }^{2}\left(u\right)-u^{2}-i\epsilon }.$$

This integral can be cast as the function in the integrand evaluated at a given point plus a principal part, but, since in evaluating Cauchy principal values, it is typical to include a small parameter, we stick to the complex representation given by (38), which already gives both the imaginary and real parts of the result.

For concreteness, we note that in the isotropic case this function can be calculated easily in terms of relatively simple functions. In terms of its real and imaginary parts, it would read:

(39) ReF(x)=μ2b28πρmcT412+32γ4+1−1γ2cT2x2−(1−cT4x4)ln|1−cT2x2||cT2x2|−(1−γ2cT2x2)2γ4ln|1−γ2cT2x2||γ2cT2x2|

(40) ImF(x)=μ2b28ρmcT4(1−cT4x4)Θ(1−|cTx|)+(1−γ2cT2x2)2γ4Θ(1−|γcTx|),

where Θ(x) is the Heaviside step function. Note that both the real and imaginary parts of *F* are nontrivial. This means that we will have anomalous dispersion with contributions to both the effective speed of propagation of the dislons and to their decay rate.

**Figure 4:**
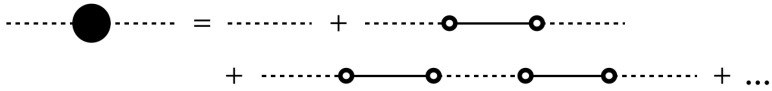
“Dressed” dislon propagator. This is the counterpart of the “dressed” phonon propagator in terms of the dislon field. This object has the advantage that it explicitly conserves energy and momentum along all dimensions in which the string is extended, whereas the exact phonon propagator only conserves energy and momentum along the dimensions that are shared with the string (time and the ${\hat{e}}_{3}$ axis).

Once we have the self-energy, we can sum over all possible insertions of phonon propagators (see Figure 4) to write the exact dislon propagator as

(41) $$S\left(\kappa ,\omega \right)=\frac{-i\hbar }{-m\omega^{2}+\Gamma \kappa^{2}-\Pi \left(\kappa ,\omega \right)},$$

which is, apart from the contractions with external phonons in the amplitude (27), all we needed to compute. With this in hand, we can now write the final result for the amplitude in a closed form. Specifically, if we define the T matrix through

(42) $$\langle f|i\rangle \equiv i{\left(2\pi \right)}^{2}\;\delta \left(\omega_{\iota }\left(k\right)-\omega_{\iota^{\prime }}\left(k^{\prime }\right)\right)\;\delta^{\left(1\right)}\left(k_{3}-k_{3}^{\prime }\right)\;T,$$

then we obtain

(43) $$T=\frac{E^{*}\left(k;\iota \right)E\left(k^{\prime };\iota^{\prime }\right)}{-\left(1+F\;\left(\frac{k_{3}}{\omega }\right)\right)\omega^{2}+\frac{\Gamma }{m}k_{3}^{2}},$$

where we have omitted the free theory result, i.e., a pure phonon propagator, as it does not represent a scattering process. Having established the form of the phonon-to-phonon scattering amplitude, we can now compute the scattering cross-section of phonons due to the presence of a dislocation line. However, before doing that, we will explore some aspects of this result to build some intuition on the physics behind it.

### 4.2. A Look at the Dislon Dispersion Relation

The exact dislon propagator (41), besides its relevance for computing phonon scattering amplitudes, also gives us the possibility to study the dispersion relation of the modes that propagate along the dislocation line directly. Indeed, the physical “asymptotic” states in a scattering picture, which are made of a superposition of “on-shell” states, are usually characterized by a dispersion relation ω(κ) determined by the (possibly complex) poles of the propagator. In the absence of a medium with which to interact, the dislon dispersion relation is defined by

(44) $$-m\omega^{2}+\Gamma \kappa^{2}=0\Rightarrow \omega^{2}\left(\kappa \right)=\frac{\Gamma }{m}\kappa^{2}.$$

This means, for example, that perturbations with frequency ω will propagate along the dislocation line with a wavenumber given by $\kappa =\pm \sqrt{m\omega^{2}/\Gamma }$, corresponding to the usual picture of wave propagation on a string.

**Figure 5:**
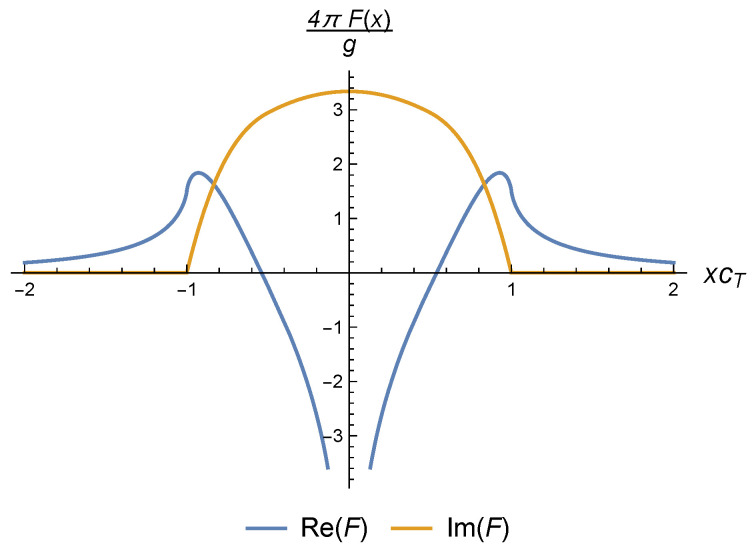
Real and Imaginary parts of the function *F*, which determines the self-energy of the dislons through $\Pi =m\omega^{2}F$ in the isotropic case. They do not vanish simultaneously. The plots were generated setting γ=2.

However, the presence of a non-trivial self-energy Π(κ,ω) complicates this picture (see Figure 5 for a typical plot of the function *F*). Even more so, the explicit expressions for the isotropic case (39), (40) are not analytic because of a branch cut on the real $x^{2}$ axis that may be seen from (38), which, although it introduces a singularity, does not give a straightforward pole structure wherein to identify “particles”. Indeed, the branch cut singularity is a reflection that the intermediate dislon states can decay to (physical) on-shell phonons, and thus we do not expect those states to be able to survive for arbitrarily long times.

It is therefore useful to distinguish the role of dislons as physically-propagating objects from their role as scatterers. While both are determined by the same exact propagator (41), the values of *x* in (38) that become relevant to observables are starkly different: in the former, *x* is fixed by the dispersion relation, whereas, in the latter, *x* is a function of the angle at which the scattering phonon is incident on the dislocation.

We study dislons as physically-propagating objects in detail in Appendix A.1, finding that unless the coupling constant

(45) $$g\equiv \frac{4\pi }{2\left(1+\gamma^{-4}\right)\ln\left(\delta /\delta_{0}\right)}$$

is greater than some critical value $g_{c}$ (which is a function of γ), the poles of the propagator *S* (41), represented by a dispersion relation ω(κ), are entirely imaginary and therefore represent purely decaying solutions, or *evanescent* waves.

If *g* were greater than this critical value $g_{c}$, propagating solutions re-appear because on-shell dislons can no longer decay to on-shell phonons because of energy and momentum conservation. That is to say, dislons are stable excitations (*particles*) in this regime. Note, however, that a typical value for $g_{c}\approx 3.44$, obtained by taking γ=2, corresponds to short- and long-distance cutoffs obeying $\delta \approx 5.6\delta_{0}$. Therefore, in a typical situation where we assume that these cutoffs are separated by an order of magnitude, we do not expect to see $g>g_{c}$, and expect mostly decaying dislon modes.

On the other hand, as we previously mentioned, one can also interpret the result for the exact dislon propagator (41) from the point of view of the scattering amplitude (43). Indeed, we can write

(46) $$T=mE^{*}\left(k;\iota \right)E\left(k^{\prime },\iota^{\prime }\right)\;\frac{i}{\hbar }S\left(k_{3},\omega \right),$$

where *S* is fully determined by the dislon dispersion relation; in fact, it is basically the inverse of the operator that determines the exact equation of motion (in Fourier space) for dislons propagating along the dislocation line after integrating out the other degrees of freedom in the system (phonons).

This is a natural point to make contact with previous studies on phonon scattering by dislocations. It has long been recognized [37,38,39] that a classically described (i.e., non-quantum) moving dislocation will experience a drag force because of the DPI (a “phonon wind”). Within the description that we have adopted in the present paper, this amounts to supplementing the string dynamics that follow from the action (3,4) with a phenomenological term to obtain

(47) $$m\ddot{X}\left(s,t\right)+B\dot{X}\left(s,t\right)-\Gamma X^{\prime \prime }\left(s,t\right)=F\left(s,t\right),$$

where *B* is a phenomenological drag parameter and *F* is the Peach–Koehler force. In particular, in the context of [30], a first-order computation of phonon scattering in perturbation theory was carried out to determine the observable effects of this damping parameter *B*, leaving it as an adjustable quantity. Since it is a first-order computation, the dislon propagator connecting the “external” phonons (in a diagrammatic sense) is correspondingly given by

(48) $$S\left(\kappa ,\omega \right)=\frac{-i\hbar }{-m\omega^{2}-iB\omega +\Gamma \kappa^{2}}.$$

Note that we have written this last dislon propagator in its quantum version (including *ℏ* in the numerator) because ultimately whatever dissipative mechanism that one observes in a macroscopic, classical limit must emerge from a quantum-mechanical description of how dislocations interact with their surroundings. Furthermore, as long as anharmonicities are small compared to the quadratic DPI, the Heisenberg equation of motion for the dislon field operator can be put in direct correspondence with the classical equation of motion for the dislon field through the identification of Poisson brackets and commutators with each other, enabling us to write (48) as a propagator for the quantum excitations associated with the LHS of Equation (47).

It is then natural to try and obtain a quantitative estimate for *B* from our explicit results for Π, the dislon self-energy. If we interpret the real part of *F* as a term that renormalizes the speed at which dislons propagate through the dislocation line at different wavelengths, then we may simply read off

(49) −iBω=−iIm{Π(κ,ω)}.

Because of the optical theorem, the imaginary part of Π will be nonzero only for values of x=κ/ω that allow for a decay to phonon states conserving momentum along the ${\hat{e}}_{3}$ direction, as well as frequency/energy ω. This means that we can focus on the region where $|x|<c_{T}^{-1}$. To get an order-of-magnitude estimate, we may take x=0, where we have, in the isotropic case,

(50) Im{Π}=ω2μ2b28ρcT41+γ−4,

implying

(51) $$B≲\frac{1+\gamma^{-4}}{8}\rho b^{2}\times \omega ,$$

where ≲ means that the phenomenological value of *B* should be less than the RHS of (51), depending on the angle of incidence of the phonon with respect to the dislocation line, but of the same order of magnitude.

While, on the one hand, this means that *B* depends on the frequency of the incident phonon scattering off the dislocation line, this also provides a quantitative estimate that can be tested by comparing with experiments that intend to probe and characterize the equation of motion for dislocations phenomenologically, as with (47).

Finally, it is important to note that this estimate relied on using the exact dissipation rate computed from dislons propagating on an infinite string. However, experiments testing this result may be sensitive to the length of the dislocation line *L*, which undoubtedly yields a different notion of dislon self-energy because momentum along the ${\hat{e}}_{3}$ axis is no longer conserved [17] as the exact equation of motion becomes infinitely coupled between the different modes of the string, and the identification of *B* with the imaginary part of the inverse propagator needs to be revisited. In this case, one possibility for a direct identification would be to simply identify *B* with the decay rate of a given dislon mode, probably corresponding to the first normal mode of the string. Additional effects to consider in order to make contact with experimental results should include a non-vanishing mean dislocation velocity, the effect of cubic and higher order terms in the phonon–dislon interaction and the effect of a finite temperature. Although it should be possible to tackle these phenomena within the formalism we present, doing so is outside the scope of the present work.

The preceding discussion, that is, the identification of a decay rate from the imaginary part of the self-energy Π, is tantamount to quantifying the width of the resonance peak when a phonon scatters off a dislocation, as long as the coupling *g* is “weak” (g≪1), and so the “free” kinetic terms dominate. On the other hand, at large coupling g≫1, the self-energy Π becomes large and there is no obvious notion of a “resonance”, since the virtual dislons in the amplitude will never be close to being “on-shell” in the sense of the free theory. In this situation, it wouldn’t be possible to infer *B* from a “resonance” peak in the phonon-to-phonon amplitude. We present a more detailed discussion on these resonances of the dislon propagator in Appendix A.2.

### 4.3. The Phonon Propagator in the Presence of a Single Dislocation

**Figure 6:**
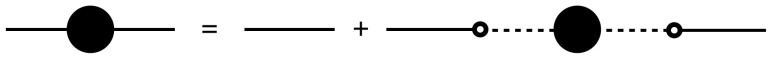
“Dressed” phonon propagator in terms of the exact dislon propagator.

Just as we derived the exact dislon propagator (41), we can similarly derive the exact (or “dressed”) time-ordered phonon propagator by computing the sum of diagrams in Figure 2. This is most easily done by taking the expression for the exact dislon propagator and using it to compute the phonon propagator, as is shown in Figure 6. Alternatively, one could take the scattering amplitude (43) and “restore” the external propagators that would be in the place of the creation and annihilation operators in the original amplitude of interest (27), in the way that the LSZ reduction formula connects correlation functions and scattering amplitudes.

Either way, one obtains that the time-ordered phonon propagator is given by

(52) $$\begin{array}{cc} \frac{i}{\hbar }G_{ij}\left(k,\omega ;k^{\prime },\omega^{\prime }\right) & ={\left(2\pi \right)}^{4}\delta \left(\omega -\omega^{\prime }\right)\delta^{3}\left(k-k^{\prime }\right)\sum_{\iota }\frac{\varepsilon_{\iota }{\left(\hat{k}\right)}_{i}\varepsilon_{\iota }^{*}{\left(\hat{k}\right)}_{j}}{-\rho \omega^{2}+\rho c_{\iota }^{2}k^{2}-i\epsilon }\\ & \;\;\;\;+{\left(2\pi \right)}^{2}\delta \left(\omega -\omega^{\prime }\right)\delta \left(k_{3}-k_{3}^{\prime }\right)\sum_{\iota }\frac{\varepsilon_{\iota }{\left(\hat{k}\right)}_{i}\varepsilon_{\iota }^{*}{\left(\hat{k}\right)}_{i^{\prime }}M_{i^{\prime }l}k_{l}}{-\rho \omega^{2}+\rho c_{\iota }^{2}k^{2}-i\epsilon }\\ & \;\;\;\;\;\;\;\;\times \frac{b^{2}N^{2}}{-m\omega^{2}+\Gamma k_{3}^{2}-\Pi \left(k_{3},\omega \right)}\sum_{\iota^{\prime }}\frac{k_{l^{\prime }}^{\prime }M_{j^{\prime }l^{\prime }}\varepsilon_{\iota^{\prime }}{\left({\hat{k}}^{\prime }\right)}_{j^{\prime }}\varepsilon_{\iota^{\prime }}^{*}{\left({\hat{k}}^{\prime }\right)}_{j}}{-\rho \omega^{\prime 2}+\rho c_{\iota^{\prime }}^{2}k^{\prime 2}-i\epsilon } \end{array}$$

where we have included the energy-momentum conservation Dirac deltas explicitly because the DPI does not conserve momentum in the plane orthogonal to the dislocation line.

In principle, one could diagonalize the continuous matrix $G_{ij}\left(k,\omega ;k^{\prime },\omega^{\prime }\right)$ (with “rows” and “columns” given by (k,ω) and $\left(k^{\prime },\omega^{\prime }\right)$) to obtain “renormalized quasi-phonons” [21] that define the eigenstates of the system after integrating out the dislon degrees of freedom, and then compute thermal transport properties by using linear response theory on this new basis. In this picture, “quasi-phonons” reproduce the “free” phonon spectrum in the limit where the DPI becomes negligible, whereas, for arbitrary non-zero strength of the DPI, these “quasi-phonons” will exhibit a renormalized dispersion relation that depends on the DPI dynamics.

While this is certainly an interesting and ultimately necessary direction to explore if one wants to have a full understanding of what the spectrum of extended excitations inside the solid is, our goals for the remainder of this work will be limited to establishing how a macroscopic thermal transport anisotropy will be generated because of the presence of dislocations in a framework that is easy to extend and apply to the quantitative analysis of thermal conductivity measurements for different distributions of dislocations. In this latter sense, the reason why we do not presently study the phonon spectrum from Equation (52) is because that expression for the propagator is still insufficient to describe a solid threaded by many dislocation lines, where we should include contributions coming from the dislon excitations on every dislocation line threading the solid, located at arbitrary positions ${\left\{\left(x_{n},y_{n},s\right)\right\}}_{n}$ extended along the ${\hat{e}}_{3}$ axis. An adequate diagonalization to obtain the vibrational eigenstates of the system should thus incorporate all of these contributions. Therefore, unless additional assumptions regarding the distribution of dislocation positions are provided, it seems comparatively unwieldy to engage in this computation using the “quasi-phonon” approach when compared to using the original phonon basis for the excitations in the solid. We emphasize that, since the degrees of freedom of the system are the same regardless of the basis of states chosen to study it (phonons or “quasi-phonons”), we can choose either of them to compute the thermal transport properties of the system.

Instead, we will follow a more old-fashioned approach to compute thermal transport properties in a solid threaded by many dislocations, where we transition from a quantum-mechanical amplitude to a macroscopic thermal conductivity by studying phonon transport in a relaxation time approximation (RTA) by calculating the 1-to-1 phonon transition rates that the phonon propagator (52) implies, and then using this lifetime to write down a thermal conductivity as is done in kinetic theory [40]. Concretely, these transition rates are captured by the second term in (52), or, equivalently, through the scattering amplitude (43).

Our approach notwithstanding, we consider that the propagator (52), and its real-time thermal field theory counterparts deserve special attention in a separate study devoted to establishing the impact of this theory of DPI on thermal conductivity through a Kubo formula in linear response theory, which the authors hope to undertake in the near future.

## 5. Implications on Thermal Transport

We now turn to examining how the scattering mechanism provided by dislocations affects energy transport in a solid. In particular, we focus on thermal transport through phonons, and set the groundwork for the computation of thermal conductivities in a solid threaded by highly-oriented dislocations. In this section, we start by discussing conventional cross-sections in a scattering picture, and then move on to consider the lifetime of phonons participating in thermal transport. Finally, we compare with the original work of Klemens [9] and Carruthers [16] and study the anisotropy in thermal transport that arises due to a large number of long dislocations threading the solid.

### 5.1. The Scattering of a Phonon by a Dislocation: Cross Sections

From (43), the differential scattering cross section from mode (k,ι) to mode $\left(k^{\prime },\iota^{\prime }\right)$ is given directly by taking the absolute value squared of the scattering amplitude T, integrating over the length of the wave-vector k=|k|, and dividing by the incident flux $v_{\iota }^{g}\left(\hat{k}\right)/V$ times the norm of the incident state *V* (where *V* is the volume of the elastic continuum, and $v_{\iota }^{g}\left(\hat{k}\right)=\left|d\omega_{\iota }/dk\right|$ is the group velocity of sound waves in the elastic medium). One obtains

(53) $$\frac{d\sigma_{\iota \iota^{\prime }}}{d\Omega }=\frac{L\delta (k_{3}-k_{3}^{\prime })}{2\pi c_{\iota^{\prime }}^{5}\left({\hat{k}}^{\prime }\right)c_{\iota }^{2}\left(\hat{k}\right)v_{\iota }^{g}\left(\hat{k}\right)}{\left(\frac{N^{2}b^{2}}{2\rho m}\right)}^{2}\frac{|{\hat{k}}_{l}M_{kl}\varepsilon_{\iota }{\left(k\right)}_{k}{|}^{2}{\left|{\hat{k}}_{l^{\prime }}^{\prime }M_{k^{\prime }l^{\prime }}\varepsilon_{\iota^{\prime }}{\left(k^{\prime }\right)}_{k^{\prime }}\right|}^{2}}{{\left|\frac{\Gamma k_{3}^{2}}{m\omega^{2}}-\left(1+F\;\left(\frac{k_{3}}{\omega }\right)\right)\right|}^{2}},$$

where *L* is the length of the dislocation, which we take to be large so that the approximation L∼(2π)δ(κ−κ)=(2π)δ(0) is justified.

Integrating over the possible outgoing states, i.e., over the relative angle dΩ between k and $k^{\prime }$, the total cross section for the mode (k,ι) may be written as

(54) $$\sigma_{\iota }\left(k\right)=\frac{L}{2c_{\iota }^{2}\left(\hat{k}\right)v_{\iota }^{g}\left(\hat{k}\right)}{\left(\frac{N^{2}b^{2}}{2\rho m}\right)}^{2}\frac{|{\hat{k}}_{l}M_{kl}\varepsilon_{\iota }{\left(k\right)}_{k}{|}^{2}}{{\left|\frac{\Gamma k_{3}^{2}}{m\omega^{2}}-\left(1+F\;\left(\frac{k_{3}}{\omega }\right)\right)\right|}^{2}}\sum_{\iota^{\prime }}\int_{-1}^{1}du\left(1-u^{2}\right)\frac{|\varepsilon_{\iota^{\prime }}^{xy}{\left(u\right)|}^{2}}{c_{\iota^{\prime }}^{5}\left(u\right)}\delta \;\left(k_{3}-\frac{\omega u}{c_{\iota^{\prime }}\left(u\right)}\right),$$

and, averaging over dislocation orientations (Burgers vector) in the ${\hat{e}}_{1}-{\hat{e}}_{2}$ plane, one obtains

(55) $$\begin{array}{cc} {\bar{\sigma }}_{\iota }\left(k\right)= & \;\frac{L}{4\omega_{\iota }\left(k\right)c_{\iota }^{2}\left(\hat{k}\right)v_{\iota }^{g}\left(\hat{k}\right)}{\left(\frac{N^{2}b^{2}}{2\rho m}\right)}^{2}\frac{|\varepsilon_{\iota }^{xy}{\left(\cos\theta \right)|}^{2}\sin^{2}\theta }{{\left|\frac{\Gamma k_{3}^{2}}{m\omega^{2}}-\left(1+F\;\left(\frac{k_{3}}{\omega }\right)\right)\right|}^{2}}\\ & \times \sum_{\iota^{\prime }}\int_{-1}^{1}du\left(1-u^{2}\right)\frac{|\varepsilon_{\iota^{\prime }}^{xy}{\left(u\right)|}^{2}}{c_{\iota^{\prime }}^{5}\left(u\right)}\left|\frac{c_{\iota^{\prime }}^{2}\left(u\right)}{c_{\iota^{\prime }}\left(u\right)-uc_{\iota^{\prime }}^{\prime }\left(u\right)}\right|\delta \;\left(u-u_{\iota^{\prime }}\left(k_{3}/\omega \right)\right), \end{array}$$

where $\cos\theta =\hat{k}\cdot {\hat{e}}_{3}$, $u_{\iota }\left(x\right)$ is defined as the solution to the equation $u=xc_{\iota }\left(u\right)$, and $c_{\iota }^{\prime }\left(u\right)\equiv dc_{\iota }\left(u\right)/du$.

We can proceed further without overcomplicating the expressions if we assume an isotropic elastic continuum because here we only have two sound speeds, $c_{T}$ (×2) and $c_{L}$, corresponding to transverse and longitudinal polarizations that do not depend on the direction of propagation θ. Moreover, the sum over transverse polarizations can be evaluated (for an isotropic medium) to

(56) $$\sum_{\iota =T_{1},T_{2}}{\left|\varepsilon_{\iota }^{xy}\left(u\right)\right|}^{2}=1+u^{2},$$

whereas, for longitudinal polarization, we have

(57) $$|\varepsilon_{L}^{xy}{\left(u\right)|}^{2}=1-u^{2}.$$

The integral over *u* is now straightforward, as the Dirac delta becomes $\delta (u-c_{\iota^{\prime }}k_{3}/\omega )$. It gives (in a strictly isotropic elastic continuum)

(58) $$\begin{array}{cc} & \sum_{\iota^{\prime }}\int_{-1}^{1}du\left(1-u^{2}\right)\frac{|\varepsilon_{\iota^{\prime }}^{xy}{\left(u\right)|}^{2}}{c_{\iota^{\prime }}^{5}\left(u\right)}\left|\frac{c_{\iota^{\prime }}^{2}\left(u\right)}{c_{\iota^{\prime }}\left(u\right)-uc_{\iota^{\prime }}^{\prime }\left(u\right)}\right|\\ & \;\;\;\;\times \delta \;\left(u-u_{\iota^{\prime }}\left(k_{3}/\omega \right)\right)\\ & \;\;=\frac{1}{c_{T}^{4}}\left(1-{\left(\frac{c_{T}k_{3}}{\omega }\right)}^{4}\right)\Theta \left(1-\left|\frac{c_{T}k_{3}}{\omega }\right|\right)+\frac{1}{c_{L}^{4}}{\left(1-{\left(\frac{c_{L}k_{3}}{\omega }\right)}^{2}\right)}^{2}\Theta \left(1-\left|\frac{c_{L}k_{3}}{\omega }\right|\right)\\ & \;\;\equiv \frac{1}{c_{T}^{4}}I\left(k_{3}/\omega \right). \end{array}$$

For physical phonons, the first Heaviside function is always one because $\omega =c_{\iota }k\Rightarrow \left|c_{T}k_{3}/\omega \right|<1$. To cast everything in terms of dimensionless functions, we can define

(59) $$A\left(k_{3}/\omega \right)\equiv {\left|\frac{\Gamma k_{3}^{2}}{m\omega^{2}}-\left(1+F\;\left(\frac{k_{3}}{\omega }\right)\right)\right|}^{2},$$

which captures the polarization-independent contribution that depends on $k_{3}/\omega$. We can also work through the $N^{2}b^{2}/\left(2\rho m\right)$ factor in the isotropic limit, where N=μ and $m=\rho b^{2}\left(1+\gamma^{-4}\right)\ln\left(\delta /\delta_{0}\right)/\left(4\pi \right)$, giving

(60) $${\left(\frac{N^{2}b^{2}}{2\rho m}\right)}^{2}=c_{T}^{8}{\left(\frac{2\pi }{\left(1+\gamma^{-4}\right)\ln\left(\delta /\delta_{0}\right)}\right)}^{2}\equiv c_{T}^{8}g^{2},$$

where, as before, we have introduced the dimensionless coupling constant *g* for notational simplicity. In this form, the cross-sections of a phonon scattering by a single dislocation read

(61) $$\sigma_{T}\left(k\right)=\frac{Lc_{T}g^{2}}{8\omega_{T}\left(k\right)}\left(1-\cos^{4}\left(\theta \right)\right)\frac{I(k_{3}/\omega_{T}\left(k\right))}{A(k_{3}/\omega_{T}\left(k\right))}$$

and

(62) $$\sigma_{L}\left(k\right)=\frac{Lc_{T}g^{2}}{4\gamma^{3}\omega_{L}\left(k\right)}\sin^{4}\left(\theta \right)\frac{I(k_{3}/\omega_{L}\left(k\right))}{A(k_{3}/\omega_{L}\left(k\right))},$$

where we have averaged over the two polarizations in the transverse case.

### 5.2. The Scattering of a Phonon by a Dislocation: Lifetimes

We now turn to the task of estimating the phonon lifetime in thermal transport due to scattering by dislocations. For simplicity, we shall assume that the elastic continuum is isotropic, and will consider $\Lambda_{d}$ parallel dislocations per unit area. This is a slightly different calculation to that of the cross-section because in writing down an equation for the evolution of the expected occupancy of mode (k;ι), we need to include transition probabilities both *from* and *to* any other mode in the theory. Our goal will be to calculate the single-mode phonon decay rates $\tau_{\iota }^{-1}\left(k\right)$, so that they may later be used to compute the thermal conductivity tensor using the relation [40]

(63) $$\begin{array}{cc} K_{ij}=\sum_{\iota }\;\int \;\;\frac{d^{3}k}{{\left(2\pi \right)}^{3}} & \frac{e^{\hbar \omega_{\iota }\left(k\right)/k_{B}T}}{{\left(e^{\hbar \omega_{\iota }\left(k\right)/k_{B}T}-1\right)}^{2}}{\left(\frac{\hbar \omega_{\iota }\left(k\right)}{k_{B}T}\right)}^{2}k_{B}\tau_{\iota }\left(k\right){\left[v_{\iota }\left(k\right)\right]}_{i}{\left[v_{\iota }\left(k\right)\right]}_{j}, \end{array}$$

where $v_{\iota }\left(k\right)$ is the phonon velocity of propagation for the mode (k;ι). In a sense, this is a relaxation time approximation because Equation (63) assumes that all transport phenomena can be described through a single phonon lifetime $\tau_{\iota }\left(k\right)$ for each mode separately.

**Figure 7:**
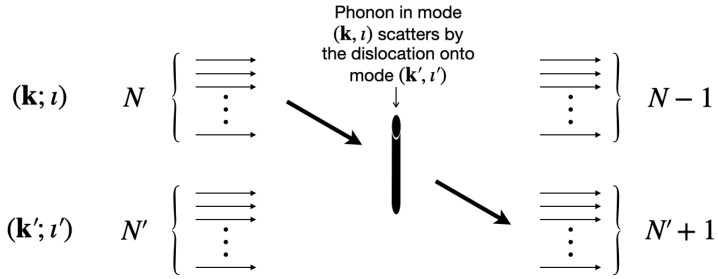
Diagrammatic illustration of the process that gives the rate at which phonons populating the mode (k;ι) transition to $\left(k^{\prime };\iota^{\prime }\right)$.

The scattering processes that contribute to this phonon lifetime may be illustrated as in Figure 7: one of the phonons of mode (k;ι) scatters off the dislocation line, and goes into the mode $\left(k^{\prime },\iota^{\prime }\right)$. Out of these individual processes, we want to first determine the rates of transition between the different modes, and then write down the full lifetime of mode (k;ι) by subtracting the rate at which phonons are created in this mode with the rate at which they decay.

The derivation of the lifetimes proceeds as follows: since the interaction under consideration couples an ingoing (k,ι) mode with an outgoing $\left(k^{\prime },\iota^{\prime }\right)$, the relevant amplitude admits the following schematic representation in terms of harmonic oscillator ladder operators (*a* and $a^{\prime }$ for the modes (k;ι) and $\left(k^{\prime };\iota^{\prime }\right)$, respectively)

(64) $$\langle f|T_{1-1}a{\left(a^{\prime }\right)}^{†}|i\rangle$$

with

(65) $$|i\rangle =\frac{{\left(a^{†}\right)}^{N}}{N!}\frac{{\left({\left(a^{\prime }\right)}^{†}\right)}^{N^{\prime }}}{N^{\prime }!}\left|0\rangle =\right|N,N^{\prime }\rangle$$

and

(66) $$|f\rangle =\frac{{\left(a^{†}\right)}^{N-1}}{(N-1)!}\frac{{\left({\left(a^{\prime }\right)}^{†}\right)}^{N^{\prime }+1}}{(N^{\prime }+1)!}\left|0\rangle =\right|N-1,N^{\prime }+1\rangle ,$$

where $T_{1-1}$ represents the one-to-one particle transition amplitude: it is essentially a placeholder for the phonon-to-phonon scattering amplitude in (43). Standard algebra in quantum mechanics then gives

(67) $$\langle f|T_{1-1}a{\left(a^{\prime }\right)}^{†}|i\rangle =T_{1-1}\sqrt{N(N^{\prime }+1)},$$

implying that the transition rate is proportional to $|T_{1-1}{|}^{2}N\left(N^{\prime }+1\right)$. Conversely, the rate of transition from mode $\left(k^{\prime },\iota^{\prime }\right)$ to (k;ι) is proportional to $|T_{1-1}{|}^{2}N^{\prime }\left(N+1\right)$.

Let us stress that, in the above discussion, we have assumed that the transitions of interest involve only one scattering process at the same time, but, as opposed to what one would do in leading-order perturbation theory, we keep the full one-to-one phonon interaction amplitude, which accounts for all the scattering dynamics of a single phonon. Including simultaneous transition processes is feasible within the framework presented in this work, but it falls outside the scope of the present approximation, in which only single-mode lifetimes are considered.

If we now assume the number distribution *N* can be written as an equilibrium distribution $N_{0}$ plus a deviation *n*, i.e., $N=N_{0}+n$, then the time derivative of the occupancy of mode (k;ι) is proportional to

(68) $$\begin{array}{cc} \sum_{\left(k^{\prime };\iota^{\prime }\right)}{\left|T\right|}^{2}\left(\left(N+1\right)N^{\prime }-N\left(N^{\prime }+1\right)\right) & ={\left|T\right|}^{2}\left(N^{\prime }-N\right)\\ & ={\left|T\right|}^{2}\left(n^{\prime }-n\right), \end{array}$$

where we have assumed that the equilibrium distribution $N_{0}$ is the same for all phonon modes. This is indeed the case if said equilibrium distribution is the Bose–Einstein distribution. In particular, if we have $\Lambda_{d}$ parallel dislocations per unit area, this means that the relaxation time for mode (k,ι), which can be written as $\tau_{\iota }{\left(k\right)}^{-1}=-{\dot{n}}_{\iota ,k}/n_{\iota ,k}$, is given by

(69) $$\tau_{\iota }{\left(k\right)}^{-1}=\frac{\Lambda_{d}v_{\iota }^{g}\left(\hat{k}\right)}{L}\sum_{\iota^{\prime }}\int d\Omega \frac{d\sigma_{\iota \iota^{\prime }}}{d\Omega }\left(1-\frac{n_{\iota^{\prime }}}{n_{\iota }}\right),$$

where $d\sigma_{\iota \iota^{\prime }}/d\Omega$ is given by (53), the differential cross section in vacuum.

In the presence of a temperature gradient, the out-of-equilibrium occupation numbers $n_{\iota }$, $n_{\iota^{\prime }}$ should reflect the fact that heat is being transported along a fixed direction. Following the works of Klemens [9] and Carruthers [16], we use the estimate

(70) $$n_{\iota ,k}\propto \hat{k}\cdot \nabla T,$$

in the spirit that the out-of-equilibrium distribution will imply a heat current in the direction defined by ∇T. We leave the examination of this assumption from a more modern perspective of thermal transport using linear response coefficients [41] in thermal quantum field theory (as recently suggested [22]) for future work.

There are two main cases of interest: thermal transport parallel to the dislocation lines and perpendicular to them. The first case is a direct extension of the cross-sections we computed in the previous section, as

(71) $$\nabla T\|{\hat{e}}_{3}\Rightarrow \frac{n_{\iota^{\prime }}}{n_{\iota }}=d_{\iota^{\prime }\iota }\frac{\cos(\theta_{\mathrm{out}})}{\cos(\theta_{\mathrm{in}})},$$

where $d_{\iota \iota^{\prime }}$ is unity if $\iota =\iota^{\prime }$ (because the proportionality constant in (70) is the same), and a number to be determined if $\iota \neq \iota^{\prime }$, satisfying $d_{\iota \iota^{\prime }}={\left(d_{\iota^{\prime }\iota }\right)}^{-1}$. Because both $k_{3}$ and ω are conserved for transverse-to-transverse as well as for longitudinal-to-longitudinal scattering, in these situations, this factor is equal to one, and therefore these processes do not contribute to the phonon lifetime.

In principle, transverse to longitudinal and vice versa processes could contribute. Note that, because both ingoing and outgoing scattering angles satisfy $\cos\theta =k_{3}/\left|k\right|=c_{\iota }k_{3}/\omega$, the conservation of $k_{3}/\omega$ implies that $c_{\iota }^{-1}\cos\left(\theta \right)$ is also conserved amongst ingoing and outgoing modes. Therefore,

(72) $$\frac{\cos\theta_{T}}{c_{T}}=\frac{\cos\theta_{L}}{c_{L}}\Rightarrow \gamma \cos\theta_{T}=\cos\theta_{L},$$

meaning that longitudinal polarization can always scatter to transverse polarization, but for some angles transversely polarized phonons cannot scatter onto longitudinal modes.

Now comes a crucial observation: since we expect a steady current to be held in place, a condition which is part of the definition of $n_{\iota ,k}$, all phonon lifetimes must be positive (if they were negative, it means that one particular mode continues to receive phonons from another mode perpetually). Since the kinematics of the phonon-to-phonon scattering process fix the angle of the outgoing phonon relative to the dislocation line θ, the quotient $\cos\left(\theta_{\mathrm{out}}\right)/\cos\left(\theta_{\mathrm{in}}\right)$ can be either γ or 1/γ, and therefore the sign of $\tau_{\iota }{\left(k\right)}^{-1}$ is fixed by $1-n_{\iota^{\prime }}/n_{\iota }$. However, because $\tau_{\iota }^{-1}$ is positive, we must have

(73) $$\frac{n_{T}}{n_{L}}=\frac{d_{TL}}{\gamma }\leq 1,$$

for the longitudinal-to-transverse transition ratio, and

(74) $$\frac{n_{L}}{n_{T}}=d_{LT}\gamma \leq 1$$

for the transverse-to-longitudinal ratio. This implies that $d_{TL}=\gamma =d_{LT}^{-1}$ because otherwise the kinematically allowed processes would drive the thermal current out of its steady state.

Therefore, we have that

(75) $$\tau_{T}^{\|}{\left(k\right)}^{-1}=0,$$

(76) $$\tau_{L}^{\|}{\left(k\right)}^{-1}=0.$$

Therefore, thermal transport in the direction parallel to the dislocation can only be impeded by scattering mechanisms that are not due to dislocations, at least directly.

The other case of interest is to take the temperature gradient perpendicular to the dislocation line. For definiteness, we take the temperature gradient to be oriented along a line on the ${\hat{e}}_{1}-{\hat{e}}_{2}$ plane, defined by an angle $\phi_{\nabla }$

(77) $$\nabla T\|\cos\left(\phi_{\nabla }\right){\hat{e}}_{1}+\sin\left(\phi_{\nabla }\right){\hat{e}}_{2},$$

so that

(78) $${\hat{k}}^{\prime }\cdot \nabla T\propto \sin\left(\theta^{\prime }\right)\left(\cos\left(\phi^{\prime }\right)\cos\left(\phi_{\nabla }\right)+\sin\left(\phi^{\prime }\right)\sin\left(\phi_{\nabla }\right)\right).$$

with $\phi^{\prime }$ the azimuthal angle of the outgoing phonon. Now, note that the $\phi^{\prime }$-dependent piece in (53) is given entirely by $|{\hat{k}}_{l}^{\prime }M_{kl}\varepsilon_{\iota^{\prime }}{\left(k^{\prime }\right)}_{k}{|}^{2}$, which, after a brief inspection, can be shown to involve an even number of trigonometric functions $\sin(\phi^{\prime })$, $\cos{\left(\phi \right)}^{\prime }$ as factors in the integrand. Since the integral over a full period of an odd power of trigonometric functions vanishes, we conclude that the $n_{\iota^{\prime }}/n_{\iota }$ term does not contribute to the phonon lifetime. Therefore, the phonon lifetime for a temperature gradient perpendicular to the dislocation line is given by $\tau_{\iota }^{⊥}{\left(k\right)}^{-1}=v_{\iota }^{g}\left(\hat{k}\right)\Lambda_{d}\sigma_{\iota }\left(k\right)/L$. Explicitly,

(79) $$\tau_{T}^{⊥}{\left(k\right)}^{-1}=\frac{\Lambda_{d}c_{T}^{2}g^{2}}{8\omega_{T}\left(k\right)}\left(1-\cos^{4}\left(\theta \right)\right)\frac{I(\cos\left(\theta \right)/c_{T})}{A(\cos\left(\theta \right)/c_{T})},$$

and

(80) $$\tau_{L}^{⊥}{\left(k\right)}^{-1}=\frac{\Lambda_{d}c_{T}^{2}g^{2}}{4\gamma^{2}\omega_{L}\left(k\right)}\sin^{4}\left(\theta \right)\frac{I(\cos\left(\theta \right)/c_{L})}{A(\cos\left(\theta \right)/c_{L})}\;.$$

Equations (75), (76), (79), and (80) constitute our results for the phonon lifetimes in an isotropic solid threaded by infinitely long dislocations along the ${\hat{e}}_{3}$ axis.

This model has one free parameter, given by the short- and long-distance cutoff lengths through $\ln(\delta /\delta_{0})$ that appear in the theory when we idealize the dislocation as a string. Equivalently, we can take *g* to be the free parameter in this description. All other quantities can be determined from macroscopic measurements of the elastic continuum, which makes the theory rather appealing in the sense that it is not overly sensitive to the microscopic constituents of the dislocation line.

### 5.3. A Comparison with Klemens’ and Carruthers’ Models

At this point, it becomes paramount to compare these results with previous models for phonon scattering by dislocations. The Carruthers model [16], after several approximations including considering a simple cubic lattice, and only considering incident phonons perpendicular to the dislocation (which is the incident direction of maximum scattering in that model), gives a relaxation time of

(81) $$\tau^{\mathrm{Carruthers}}{\left(k\right)}^{-1}=\frac{1}{3}\left|k\right|\Lambda_{d}b^{2}G^{2}c_{s}{\left[\ln\left(b\sqrt{\Lambda_{d}}\right)\right]}^{2},$$

where *G* is the Grüneisen parameter, and $c_{s}$ is the average sound speed in the material.

Klemens’ model [9], which historically was introduced earlier, gives

(82) $$\tau_{\iota }^{\mathrm{Klemens}}{\left(k\right)}_{\mathrm{strain}\;\mathrm{field}}^{-1}\propto \omega_{\iota }\left(k\right)\Lambda_{d}b^{2}G^{2}$$

where the proportionality constant is an O(1) number that depends on the ratio of edge and screw dislocation densities, as well as on the Poisson ratio. This model also provides a phonon-dislocation scattering contribution from the cores of dislocations, which may be approximated as

(83) $$\tau_{\iota }^{\mathrm{Klemens}}{\left(k\right)}_{\mathrm{core}}^{-1}=\Lambda_{d}V_{a}^{4/3}\omega_{\iota }{\left(k\right)}^{3}/c_{s}^{2},$$

where $V_{a}$ is the volume per atom in the solid. We can now compare these results with our expressions for the phonon lifetimes, in the case where $\Lambda_{d}/L$ is the number of dislocations per unit of volume in a highly oriented array (assuming the long dislocations thread the elastic continuum from side to side).

Let us start by examining the strength of the scattering. Quick inspection of our results (79)–(80) shows that in our model the phonon lifetime scales as

(84) $$\tau^{-1}\propto \Lambda_{d}c_{s}^{2}\omega^{-1}$$

at fixed θ, with the other factors being of O(1). The fact that the scattering cross section for phonon scattering by a dynamically responding, infinitely long, dislocation scales like the inverse of the phonon frequency goes back to early results of Eshelby and Nabarro [42,43,44]. The similarities and differences with Klemens’ and Carruthers’ models are evident at this point:

1. All models (even though Klemens’ and Caurruthers’ results we have shown here do not make this explicit) have a vanishing phonon decay rate at θ=0,π, i.e., when the phonon is incident parallel to the dislocation line, favoring thermal transport in this direction over the others.
2. All models have a linear dependence on the dislocation density, with the observation that Carruthers’ model has an additional logarithmic sensitivity to the dislocation density because of how the strain field is modeled. This makes the interaction strength of Carruthers’ model generically stronger than Klemens’.
3. The other parameters that control the magnitude of the phonon lifetime are $c_{s}$, the sound speed in the material, and *b*, the dislocation’s Burgers vector. Incidentally, our model is insensitive to the value of the Burgers vector, being only dependent on the macroscopic parameters $c_{s}$ and γ.
4. In stark contrast to what both Klemens’ and Carruthers’ models predict, the phonon lifetime in our model is larger at smaller frequencies, depending on the phonon energy as $\omega^{-1}$ over the range of frequencies where the infinite dislocation line approximation holds kL≫1. In particular, this means that the thermal transport anisotropy induced by dislocations will become stronger at lower temperatures relative to Carruthers’ and Klemens’ models.

This last point may prove to be crucial in explaining the low-temperature dependence of the thermal conductivity in a material threaded by dislocations from side to side, as has been recently observed by Sun et al. in thin InN films [15], an effect that is not captured by earlier models. This will be explored quantitatively in upcoming work.

### 5.4. Thermal Transport Anisotropy

Note that one clear advantage of our result is that the angular dependence of the phonon lifetime on the polar angle θ is explicit, and therefore we can compute estimates for the anisotropy in thermal conductivity quantitatively. We proceed in the isotropic case, where we have explicit expressions for the scattering cross-sections and lifetimes. At each fixed frequency ω, the differential thermal conductivity tensor $dK_{ij}$, i.e., the contributions that the full thermal conductivity tensor $K_{ij}$ receives from modes with single-phonon energies of ℏω, may be used to study the generation of thermal transport anisotropy at each energy scale. In particular, we can write

(85) $$dK_{ij}\propto d\omega \sum_{\iota }\int \;d\Omega \;c_{\iota }^{2}{\hat{k}}_{i}{\hat{k}}_{j}\tau_{\iota }\left(k\right),$$

where we have omitted other temperature- and energy-dependent factors. Furthermore, if we use that τ only depends on the direction of propagation through the angle θ, one gets (now in matricial notation, where the first two rows/columns correspond to the ${\hat{e}}_{1}$, ${\hat{e}}_{2}$ directions and the third to ${\hat{e}}_{3}$)

(86) $$dK\propto d\omega \sum_{\iota }c_{\iota }^{2}\int_{0}^{\pi }d\theta \sin\left(\theta \right)\left[\begin{array}{ccc} \frac{\sin^{2}\left(\theta \right)}{2}\tau_{\iota }^{⊥}\left(\omega ,\theta \right) & & \\ & \frac{\sin^{2}\left(\theta \right)}{2}\tau_{\iota }^{⊥}\left(\omega ,\theta \right) & \\ & & \cos^{2}\left(\theta \right)\tau_{\iota }^{\|}\left(\omega ,\theta \right) \end{array}\right],$$

in which we have made explicit that the decay rate $\tau^{-1}$ depends only on the phonon energy and on the angle between the direction of propagation with the dislocation line axis.

With these definitions in hand, we can now calculate the anisotropy ratio $r_{\iota }$ between differential thermal conductivities (per unit frequency/energy ω and per polarization mode ι) parallel and perpendicular to the dislocation line by writing

(87) $$r_{\iota }\equiv \frac{2\int_{0}^{\pi }d\theta \sin\left(\theta \right)\cos^{2}\left(\theta \right)\tau_{\iota }^{\|}\left(\omega ,\theta \right)}{\int_{0}^{\pi }d\theta \sin^{3}\left(\theta \right)\tau_{\iota }^{⊥}\left(\omega ,\theta \right)},$$

which we shall call a *differential anisotropy ratio—DAR*, as an estimate of how large is the anisotropy in heat transport at each energy scale ℏω. As an alternative definition, the DAR represents the ratio between the angular-averaged mean free paths for phonon transport parallel to the dislocation line and perpendicular to the dislocation line, at a fixed single-phonon energy ℏω and temperature *T*. In particular, this DAR can reach arbitrarily large values if thermal transport is unimpeded along the direction parallel to the dislocation line.

This estimate is most relevant at low temperatures, where intrinsic phonon scattering due to anharmonicities of the elastic continuum becomes subdominant, and lends itself to carry out a quantitative comparison between the predictions of our dynamical approach to dislocations and the static approach of Klemens and Carruthers.

The first thing to notice is that, in our expressions due to scattering by dislocations, $\tau_{T}^{⊥}$ and $\tau_{L}^{⊥}$ diverge as $∼1/\theta^{4}$ at small polar angles (θ≪1), whereas $\tau_{T}^{\|}$ and $\tau_{L}^{\|}$ are formally infinite. This is explicit when the temperature gradient is parallel to the dislocations, as both (75) and (76) give vanishing inverse lifetimes. When the temperature gradient is perpendicular to the dislocation lines, one can see from (80) that $\tau_{L}^{⊥}\propto 1/\sin^{4}\left(\theta \right)$, and in $\tau_{T}^{⊥}$ one needs to inspect the function $I(\cos\left(\theta \right)/c_{T})$ to see that an additional factor of $\left(1-\cos^{4}\theta \right)$ appears in Equation (79). This means that, in the absence of other scattering mechanisms, both integrals in (87) are infinite because of the kinematic region where the incident phonon becomes parallel to the dislocation line. Roughly speaking, the cross-section for phonon scattering along the dislocation line vanishes. Thus, these phonons proceed unimpeded by dislocations and have an infinite relaxation time. In reality, there are mechanisms, additional to dislocation scattering, that hamper the motion of phonons along the dislocation lines and they must be considered for a realistic assessment.

These mechanisms effectively regulate the aforementioned divergence, and leave the result under quantitative control. Among these mechanisms, we highlight that:

1. there is always “intrinsic” phonon scattering due to anharmonicities in the elastic continuum,
2. the dislocation lines will usually not be perfectly aligned in a real material,
3. the consideration of finite size effects in the material introduces a boundary scattering contribution.

In what follows, we will assume that we have a perfectly aligned array of dislocations and we will neglect boundary scattering. Thus, we will only consider intrinsic phonon scattering as the dominant scattering mechanism, besides the scattering by the dislocations themselves. As noted earlier, this means that, because intrinsic phonon scattering becomes small at low temperatures, the DAR that we compute will become arbitrarily large as we decrease the temperature. In this situation, other impurity scattering mechanisms and boundary scattering will render the DAR finite at all temperatures in a real material. Nonetheless, the DAR will still become arbitrarily large as a function of frequency ω when we go to lower and lower phonon energies provided that $\omega >\omega_{1}$, with $\omega_{1}$ the lowest eigenfrequency of the dislon excitations (This behavior breaks down when ω drops below $\omega_{1}=\pi c_{\mathrm{dislon}}/L$ [17], where *L* is the length of the dislocation line and $c_{\mathrm{dislon}}$ the speed at which “free” dislons propagate on the dislocation line. As we decrease the frequency of the incident phonon further, the dislocation line will become a point-like defect from the perspective of the scattering phonons, giving a vanishing cross-section as ω→0.) because $\tau_{⊥}^{-1}$ will be dominated by dynamic dislocation scattering ∝1/ω and therefore in this regime we have $\tau_{⊥}\propto \omega$.

To combine the different decay rates, we use Matthiessen’s rule, which in our case means that

(88) $$\tau_{\iota }^{\mathrm{total}}{\left(k\right)}^{-1}=\tau_{\iota }{\left(\omega ,\theta \right)}^{-1}+\tau_{\iota }^{\mathrm{intr}}{\left(\omega ,T\right)}^{-1},$$

which is justified as long as the physical processes controlling each lifetime are independent. Geometrically, this corresponds to adding the cross-sections of the relevant scattering processes.

Now, we need estimates for the intrinsic phonon lifetime due to elastic anharmonicities. To get an order of magnitude estimate, we use the following parametrizations [45]:

(89) $$\tau_{T}^{\mathrm{intr}}{\left(\omega ,T\right)}^{-1}=B_{T}\times \omega T^{4}+B_{TU}\times \omega^{2}Te^{-C_{T}/T},$$

(90) $$\tau_{L}^{\mathrm{intr}}{\left(\omega ,T\right)}^{-1}=B_{L}\times \omega^{2}T^{3}+B_{LU}\times \omega^{2}Te^{-C_{L}/T}.$$

As a working example, we use the values reported by Asen-Palmer et al. [45] for Germanium crystals: $B_{T}=2\times 10^{-13}\;K^{-4}$, $B_{L}=2\times 10^{-21}\;s\cdot K^{-3}$, $B_{TU}=1\times 10^{-19}\;s$, $B_{LU}=5\times 10^{-19}\;s$, $C_{T}=55\;K$, and $C_{L}=180\;K$. In addition, we use $c_{T}=3000\;m/s$.

**Figure 8:**
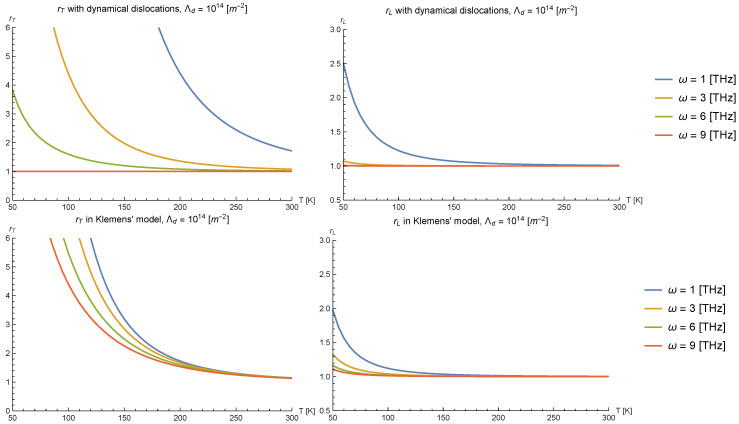
Differential anisotropy ratio $r_{\iota }$ as a function of temperature *T* for different frequencies ω. *Upper panels:* Present work, based on a dynamical response of dislocations to phonons. *Lower panels:* Klemens model, based on a static response of dislocations to phonons. *Left-hand-side panels:* Transverse polarization. *Right-hand-side panels:* Longitudinal polarization. The plots were calculated for γ=2, g=3, G=2, $b=3.5\times 10^{-10}$ m, a dislocation density of $\Lambda_{d}=10^{14}$ m${}^{-2}$, and the intrinsic phonon lifetimes parametrizations of Ge [45]. There is a significantly different anisotropy as a function of frequency between the dynamic and static cases, particularly for transverse polarization.

**Figure 9:**
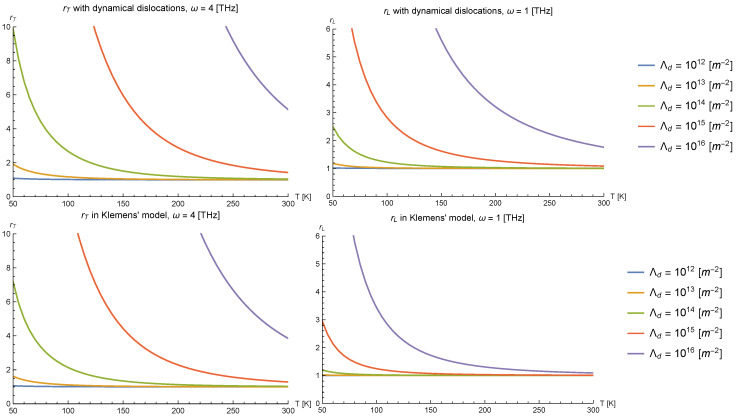
Differential anisotropy ratio $r_{\iota }$ as a function of temperature *T* for different dislocation densities $\Lambda_{d}$. *Upper panels:* Present work, based on a dynamical response of dislocations to phonons. *Lower panels:* Klemens model, based on a static response of dislocations to phonons. *Left panels:* Transverse polarization at ω=4 THz. *Right panels:* Longitudinal polarization at ω=1 THz. The plots were calculated for γ=2, g=3, G=2, $b=3.5\times 10^{-10}$ m, and the intrinsic phonon lifetime parametrizations of Ge [45]. There is no significant difference between the behavior of the static and dynamic dislocations other than the overall strength of the scattering.

We present results for the differential anisotropy ratios $r_{T}$ and $r_{L}$ at various frequencies ω as a function of temperature *T* in Figure 8 (upper panels), and at various dislocation densities $\Lambda_{d}$ in Figure 9. We chose γ=2 and g=3 as representative values for the plots. Overall, the anisotropy ratios grow as the temperature of the medium or the frequency of the incident phonons are lowered, and also grow when the dislocation density is increased, as one would qualitatively expect from the form of our phonon lifetimes. We note that the anisotropy ratio is greater for the transverse modes of phonons than for longitudinal polarization; this can be attributed to (i) that longitudinally-polarized phonons can scatter with the dislocation even if their angle of incidence is arbitrarily close to being parallel to the dislocation (with the cross section vanishing only in the strict case θ=0), making the anisotropy relatively smaller, and (ii) that their decay rate from intrinsic phonon scattering processes is larger, thus needing a larger phonon-dislocation cross-section for this process to be relevant.

To compare with Klemens’ and Carruthers’ models, we note that the inverse phonon lifetimes of both models are linear on the incident phonon energy, and therefore, qualitatively (up to a factor independent of ω), they exhibit the same behavior in the anisotropy ratios. Thus, we take Klemens’ model as a point of comparison, taking $\tau^{-1}=\omega \Lambda_{d}b^{2}G^{2}$ for both polarizations. For simplicity, we will also assume that the phonon lifetimes in the presence of a temperature gradient parallel to the dislocation line in this model are negligible. This should provide a conservative benchmark with which to decide whether the model developed herein can explain large anisotropy ratios in thermal conductivities convincingly.

We present plots for $r_{T}$, $r_{L}$ in Klemens’ model for phonon scattering in Figure 8 (lower panels). Comparing with their homologous plots in the upper panels of Figure 8, we see that, while the curves are similar for ω∼1 THz, the curves of the anisotropy ratios for other frequencies are much closer to each other in Klemens’ model than in ours. This is so precisely because of the different frequency dependence in Klemens’ model than in ours: since the phonon decay rate in Carruthers’ and Klemens’ models is linear in frequency, the anisotropy, which is generated by the difference in relative size between $\tau_{\mathrm{dislocation}}^{-1}$ and $\tau_{\mathrm{intrinsic}}^{-1}$, is less sensitive to changes in the incident phonon frequency than in our model because both decay rates grow with ω. In contrast to this linear growth in frequency, in our model, the phonon lifetime due to scattering by dislocations decreases as $\omega^{-1}$ with increasing frequency. Consequently, this makes the differential anisotropy ratio more sensitive to variations in the frequency than in Carruthers’ or Klemens’ models.

Figure 9 displays the same differential anisotropy ratios $r_{T}$ and $r_{L}$, but this time at fixed frequency and varying dislocation density. Unlike the frequency dependence of the anisotropies, which was bound to be different because of the distinct form of the phonon lifetimes in ours and Klemens’ models, their dependence on the dislocation density, illustrated by the distance between the different lines in each plot in Figure 9 for the two models, is not so different because all of the lifetimes depend linearly on the dislocation density $\Lambda_{d}$; only the overall strength of the scattering differs.

The above considerations make our model particularly promising in future attempts to explain large thermal conductivity anisotropies as the temperature is lowered from room temperature to ∼50 K because, at lower temperatures, the phonon frequencies/wavenumbers that mainly contribute to the bulk thermal conductivity of an elastic continuum are also smaller. Correspondingly, the differential anisotropy ratios $r_{\iota }$ will grow faster in the presently considered model than in Carruthers’ or Klemens’ models, precisely because the phonon decay rate due to dislocations goes as an inverse power of the frequency instead of linearly.

Finally, we wish to stress that the effects of scattering by long, dynamic dislocations can be of great relevance for thermal transport at low temperatures, where they provide the dominant scattering mechanism for phonons that propagate perpendicularly to the dislocation lines. Given that other imperfection scattering mechanisms (such as point-like defects, or possibly static dislocations as in Klemens’ or Carruthers’ models) will usually give contributions to the phonon decay rate that are increasing functions of frequency ω that vanish at ω=0, and as long as the dislocations are sufficiently elongated so that the typical incident phonon energy is larger than the lowest dislon eigenfrequency $\omega_{1}$ [17], and therefore that the phonon decay rate scales as 1/ω, the thermal transport anisotropy will be controlled purely by the ratio between scattering by dislocations and boundary scattering, of which the latter depends only on the phonon speed of propagation and the spatial extent of the material. Therefore, experimental studies of thermal conductivity in materials with long, highly-oriented dislocation arrays at low temperatures should provide a decisive test of the dynamic theory of the DPI.

## 6. Conclusions

We have considered a quantum theory of the dynamical modes of an infinitely long dislocation line, modeled as an elastic string, in interaction with phonons, which are the relevant quantum degrees of freedom at small deformations, in a continuous, homogeneous, elastic medium. The formalism holds for anisotropic media, and we have presented specific results when the medium is homogeneous. The interaction is through the well-known Peach–Koehler force exerted by a stress on a dislocation line. The quantum interaction depends on a dimensionless coupling constant that depends itself on a short-distance cutoff length at which the continuum theory ceases to be valid, and the theory is solved to all orders in said constant. Only small excursions of the dislocation line away from its equilibrium position are, however, allowed so that the interaction is quadratic. The behavior of the quanta of dislocation motion (“dislons”) is obtained, and it is revealed that there can be both unstable as well as stable dislons, depending on the strength of the coupling constant. From this information, it is possible to estimate the phonon contribution to the internal damping of dislocation motion when they are treated as classical (i.e., non quantum) strings, revealing a linear-in-frequency dependence for said damping. Equivalently, this dissipative term could be interpreted as a complex contribution to the “dislon” sound speed for the modes propagating on the string. The scattering cross-section for phonons by dislocations is obtained as an explicit function of phonon polarization, angle of incidence and frequency. In the infinite length approximation, we have considered that its dependence on frequency ω becomes rather simple: it behaves as $\omega^{-1}$.

The contribution to the scattering of phonons by dynamic dislocations is considered, especially in comparison with the classical models of phonon scattering by static dislocations of Klemens and Carruthers. In the case of a solid threaded by many parallel dislocations, we consider the ratio between the thermal conductivity per unit frequency for each polarization, in a direction parallel and perpendicular to the dislocation orientation (“differential anisotropy ratio”—DAR), as a function of temperature. Dynamic dislocations yield a DAR that is considerably more sensitive to frequency than static dislocations, raising the possibility of a quantitative understanding of recent experimental results on dislocation-induced thermal transport anisotropy because low-energy phonons are more susceptible to scattering than in previous models [9,16], and therefore it is possible to have a larger anisotropy at low temperatures.

We have used a continuum approximation. For the measurements of Sun et al. [15], where the dislocations are one micron in length, this seems a very good approximation. More generally, dislocations typically have lengths in the ten to one hundred nanometer range, where a continuum approach should provide a useful approximation as well. As mentioned in the body of the paper, and implemented explicitly through Equations (6) and (7), the theory has only one undetermined dimensionless parameter, the ratio of a long-distance to a short-distance cutoff length. Thus, it should be applicable to any crystalline material, irrespective of its microscopic structure, down to length scales of a few interatomic spacings. The other parameters that appear in the formulation we have employed are the mass density and elastic constants, and they are determined from the bulk properties. The Burgers vector, while it appears in the parameters characterizing a dislocation, cancels out in the phonon–dislon interaction, as a consequence of this interaction being completely determined by the elastic properties of the material. To repeat, while dislocations have historically played a dominant role in the plasticity properties of metal and alloys, the continuum approach we have employed in this paper should apply to other materials as well.

We have set up the description of quantum dislocation segments in a quantum field theory framework, which is well suited to include more particles and interactions (such as electrons) in a more complete description of a solid with a large dislocation density. Even though some of the results herein do not depend explicitly on *ℏ*, and therefore could be in principle obtained from an appropriate classical field description, the fundamentally quantum nature of phonons and the length scales involved in forming a dislocation beg for a low-energy quantum-mechanical description, which we have developed through this and earlier work [17]. Some purely quantum effects, such as phonon-mediated energy level transitions in a string-like dislocation line are more easily displayed when the dislocation segment is finite and cubic phonon–dislon interactions are considered, although the same transitions are possible in the presently discussed infinite dislocation segments. However, the experimental verification of such features would require a remarkable feat of dislocation engineering in order to be able to isolate the resulting signal and unequivocally attribute a discrete change in the energy of the probe to a specific transition inside the material. A theoretical derivation of a more robust signal that is unequivocally due to the quantum nature of dislocations is also a concrete long-term goal of this description.

A number of possible generalizations of the results presented in this paper suggest themselves: It should be possible to compute the effect of the third order phonon–dislon interactions, and bring in three-phonon terms. Another direction would be to replace the continuum description with a lattice. Describing phonons in a lattice is standard practice, but the description of dislons, and the corresponding coupling to phonons, would need some care. In addition, the interaction with screw dislocations, rather than edge dislocations as carried out in this work, should be straightforward. A specialization, rather than a generalization, would be to consider a two-dimensional lattice, where dislocations are point defects. This would make their description much simpler and would probably be of relevance for the study of two-dimensional materials [46,47,48].

Finally, we wish to emphasize that the formalism that has been employed in this work, in conjunction with recent previous results [17], is amenable to extensions to include anisotropy, as well as boundary effects that should make the model suitable for quantitative comparison with experimental data.

## Appendix A. Features of the Dislon Dispersion Relation

### Appendix A.1. Dislons as Particles: Evanescent and Propagating Modes

To find the modes of propagation for dislons as point-like particles, we can use the “usual” on-shell condition to find the physically-propagating modes $S^{-1}=0$, even with the explicit non-analytic expressions (39), (40), provided they are analytically-continued for arbitrary complex numbers *x* by re-deriving the result from (38). For simplicity, we proceed in the isotropic case, although we note that calculating the expressions from (38) numerically is straightforward in the general, anisotropic, case.

In the isotropic case, this continuation is given by

(A1) $$\begin{array}{cc} F(x) & =\;\frac{g}{4\pi }\;\left[\frac{1}{2}+\frac{3}{2\gamma^{4}}+\left(1-\frac{1}{\gamma^{2}}\right)c_{T}^{2}x^{2}-\left(1-c_{T}^{4}x^{4}\right)\ln\;\left(\frac{c_{T}^{2}x^{2}-1-i\epsilon }{c_{T}^{2}x^{2}-i\epsilon }\right)\right.\\ & \;\;\;\;\left.-\frac{{\left(1-\gamma^{2}c_{T}^{2}x^{2}\right)}^{2}}{\gamma^{4}}\ln\;\left(\frac{\gamma^{2}c_{T}^{2}x^{2}-1-i\epsilon }{\gamma^{2}c_{T}^{2}x^{2}-i\epsilon }\right)\right], \end{array}$$

where, for notational simplicity, we have introduced a dimensionless coupling constant *g* to replace the role of the logarithm $\ln(\delta /\delta_{0})$:

(A2) $$g\equiv \frac{4\pi }{2\left(1+\gamma^{-4}\right)\ln\left(\delta /\delta_{0}\right)}.$$

We must now find solutions to

(A3) $$\frac{2(1-\gamma^{-2})}{1+\gamma^{-4}}c_{T}^{2}x^{2}-\left(1+F(x)\right)=0,$$

which can be searched for in terms of the dimensionless variable $z\equiv c_{T}x$. Note that, in this setup, $z=c_{T}\kappa /\omega$, and, consequently, we will have a dispersion relation given by

(A4) $$\kappa \left(\omega \right)=z\left(g,\gamma \right)\frac{\omega }{c_{T}},$$

meaning that the imaginary part of *z* will determine the “attenuation length” of dislons at a fixed frequency as they propagate along the string. Conversely, one can define a dislon “lifetime” by writing ω(κ) and looking at the imaginary part of $z^{-1}$.

However, upon a closer look, one finds a surprise as one “turns on” *g* from zero: if one examines the function *F*, one sees that its imaginary part is nonzero around the “free” dispersion relation $\omega =\pm \kappa \sqrt{\Gamma /m}$, meaning the solution is no longer on the real *z*-axis. Moreover, the real $z^{2}$ axis with $0<z^{2}<1$ is precisely the branch cut of the logarithms in (A1), meaning its imaginary part is discontinuous “above” and “below” this line (this is unambiguous if one refers to (38)). Numerical analysis then shows that the propagating solution on the real $z^{2}$ axis in fact disappears for small (but nonzero) *g*, leaving the mentioned branch cut singularity in its place.

It turns out that the solutions to (A3) are found for negative $z^{2}$ as we increase *g* from 0: this can happen if *F* becomes more and more negative as $z^{2}$ approaches zero from the negative real $z^{2}$ axis. This is indeed the case: for negative $z^{2}$, *F* is a monotonously decreasing function of $z^{2}$, starting from F→0 as $z^{2}\to -\infty$ until it diverges when $z^{2}\to 0$. Therefore, for arbitrary positive *g*, there exists a solution to $S^{-1}=0$ with negative $z^{2}$, which means that the proportionality constant between ω and κ is a purely imaginary number. These are evanescent waves, meaning that the excitations of the string (dislons) will always decay into phonons in a characteristic lifetime. The lifetime of these modes is given by

(A5) $$t_{\mathrm{dislon}}\left(\kappa \right)=\left|z_{\mathrm{eva}}\right|\left(g\right){\left(c_{T}\kappa \right)}^{-1},$$

where $|z_{\mathrm{eva}}|$, the value of |z| associated with these evanescent waves, can be calculated numerically; it can take any value between 0 and *∞* as a monotonously increasing function of *g*.

Nonetheless, if we increase the value of *g* sufficiently, then propagating solutions at real $z^{2}>1$ do re-appear. These are no longer affected by the branch cut, as the logarithms are real functions in this domain because these states can no longer decay directly into physical phonons due to energy-momentum conservation. The critical value of *g* so that these solutions appear is defined by the equation

(A6) $$g>g_{c}\equiv \frac{4\pi \left(\frac{2(1-\gamma^{-2})}{1+\gamma^{-4}}-1\right)}{\frac{3}{2}-\frac{1}{\gamma^{2}}+\frac{3}{2\gamma^{4}}-\frac{{\left(1-\gamma^{2}\right)}^{2}}{\gamma^{4}}\ln\left(\frac{\gamma^{2}-1}{\gamma^{2}}\right)}.$$

For instance, if γ=2, one finds $g_{c}\approx 3.44$.

Above this value of *g*, we will find propagating solutions for dislons that travel with a “renormalized” speed

(A7) $$c_{\mathrm{dislon}}={\left|z_{\mathrm{prop}}\right|}^{-1}\left(g\right)c_{T},$$

where $|z_{\mathrm{prop}}|$ is the positive solution for *z* to (A3). As in the evanescent case, $|z_{\mathrm{prop}}|$ is a monotonously increasing function of *g*, and can become arbitrarily large as g→∞, meaning that the speed at which these modes propagate can become arbitrarily small, and thus even relatively short-wavelength dislon excitations become low-energy particles in the theory. Therefore, in the large *g* limit, there can be a great number of excited dislon modes that cannot decay into physical phonons, and only serve as intermediate states in the quantum-mechanical path integral that represents the scattering amplitude of phonons. Indeed, unless additional couplings are introduced in the theory, these modes will effectively be decoupled from the rest of the theory.

We must note, however, that, from a microscopical perspective, *g* cannot be arbitrarily large, as it is related to the logarithm of a division of cut-offs. At most, we could expect g∼10 if the cutoffs are related by $\delta ∼2\delta_{0}$.

### Appendix A.2. Resonances in the Dislon Propagator

While, as mentioned in the main text, resonances become ambiguous in the strong-coupled limit g≫1, further discussion of resonances makes perfect sense in the small $g^{2}$ limit. In this limit, the dislon self-energy is negligible except for the (small) cut-off it provides to the on-shell divergence of the free dislon propagator. The peak will be located at an incidence angle to the dislocation of $\cos^{2}\theta_{p}={\left(c_{\iota }k_{3}/\omega \right)}^{2}=c_{\iota }^{2}m/\Gamma$. For transverse incident polarization, this corresponds to

(A8) $$\cos^{2}\theta_{p,T}=\frac{1+\gamma^{-4}}{2(1-\gamma^{-2})}>\frac{1}{2},$$

which means that the cross-section will peak at a direction that is closer to ${\hat{e}}_{3}$ than to either ${\hat{e}}_{1}$ or ${\hat{e}}_{2}$. Presumably, this would give a larger thermal conductivity perpendicular to the dislocation line.

This is disfavored by experiments [15], and, moreover, it is physically suspect from the microscopic point of view, where we would need a large value of $\ln(\delta /\delta_{0})$. This is not a sensible limit because it requires δ to be many orders of magnitude greater than $\delta_{0}$, and, from the microscopic point of view, we expect (at most) 2–3 orders of magnitude (corresponding to $\ln(\delta /\delta_{0})∼8$ as an upper bound).

For completeness, we note that, for longitudinal incident polarization, the peak would be at angles corresponding to

(A9) $$\cos^{2}\theta_{p,L}=\gamma^{2}\frac{1+\gamma^{-4}}{2(1-\gamma^{-2})}>1,$$

which is geometrically impossible to attain. Only at the lowest possible value of γ, with λ≪μ, one might be able to observe a resonance in the limit where the incident phonon is collinear to the dislocation line.

The other limit, g≫1, shows no resonances in the scattering cross-section, as the imaginary and real parts of *F* never approach zero simultaneously (at least in the isotropic case; see Figure 5). In contrast, this limit leads to a suppression of the scattering amplitude when the incident phonon becomes perpendicular to the dislocation line because the function F(x) diverges logarithmically as x→0.

This last particularity is due to the fact that the dislocation segment under consideration is infinite, but pinned in its endpoints. The infinity of its extension implies an extra symmetry, which then gives momentum conservation. Then, scattering of phonons with $k_{3}=0$ by the dislocation line should excite the κ=0 mode of the string, which would correspond to a uniform translation. However, this excitation is not possible because the dislocation is pinned: the boundary conditions of the theory forbid such a process. In contrast to this, a finite dislocation segment does not imply a vanishing phonon cross-section at x=0 because $k_{3}$ need not be conserved, and, as such, other modes (with nonzero wavenumber) can be excited on the string.

## Appendix B. Thermal Equilibrium

As a check on the consistency of the formalism, we verify that the previous coupling automatically provides a mechanism with which phonons and dislons, the excitations on the dislocation line, reach thermal equilibrium.

Let us consider a single dislocation inside of a solid set in an environment at temperature *T*. Far away from the dislocation, the expectation value for the number of phonons is given by the Bose–Einstein distribution

(A10) $$\langle n_{k,\iota }\rangle =\frac{1}{e^{\hbar \omega_{\iota }\left(k\right)/k_{B}T}-1},$$

which in turn sources the states that will later scatter with the dislocation line. In thermal equilibrium, therefore, we expect that the dislon distribution $f_{d}$ at wavenumber κ be such that the rate at which a dislocation mode decays, denoted by $P_{d-\mathrm{decay}}\left(\kappa \right)$, is related to the probability per unit of time of a phonon being “absorbed” into a dislocation excitation $P_{\mathrm{ph}\to d}$ by:

(A11) $$P_{d-\mathrm{decay}}\left(\kappa \right)f_{d}\left(\kappa ,T\right)=\sum_{\iota }\int_{k}\frac{P_{\mathrm{ph}\to d}\left(k,\iota ;\kappa \right)}{e^{\hbar \omega_{\iota }\left(k\right)/k_{B}T}-1}.$$

However, dislons decay precisely into phonons through rates $P_{d\to \mathrm{ph}}$ that satisfy

(A12) $$P_{d-\mathrm{decay}}\left(\kappa \right)=\sum_{\iota }\int_{k}P_{d\to \mathrm{ph}}\left(\kappa ;k,\iota \right),$$

and, moreover, both processes (ph→d and vice versa) must contain $\delta (\omega_{\iota }\left(k\right)-\omega_{\kappa })$ as an overall factor. This implies that (A11) is actually

(A13) $$\begin{array}{cc} & f_{d}\left(\kappa ,T\right)\sum_{\iota }\int d\Omega P_{d\to \mathrm{ph}}\left(\kappa ;\omega_{\kappa }/c_{\iota }\left(\hat{k}\right),\iota \right)\\ & =\frac{1}{e^{\hbar \omega_{\kappa }/k_{B}T}-1}\sum_{\iota }\int d\Omega P_{\mathrm{ph}\to d}\left(\hat{k}\omega_{\kappa }/c_{\iota }\left(\hat{k}\right),\iota ;\kappa \right) \end{array},$$

and therefore, because the amplitudes that give rise to both probabilities/rates of decay under the angular integral sign ($P_{d\to \mathrm{ph}}$ and $P_{\mathrm{ph}\to d}$) have the same absolute value (they are mapped onto each other by time reversal, which is a symmetry of this model), we find

(A14) $$f_{d}\left(\kappa ,T\right)=\frac{1}{e^{\hbar \omega_{\kappa }/k_{B}T}-1}.$$

This implies that, if the solid is in an environment with temperature *T*, then the modes of the strings (dislocations) will also feel the same temperature.
